# Supplementary material for: Components of genetic associations across 2,138 phenotypes in the UK Biobank highlight adipocyte biology
Source: Nat Commun. 2019 Sep 6;10:4064. doi: 10.1038/s41467-019-11953-9 (PMC6731283; doi:10.1038/s41467-019-11953-9)
Supplement: Supplementary file 1 — Supplementary Information [file 41467_2019_11953_MOESM1_ESM.pdf]

Supplementary Information

Components of genetic associations across 2,138 phenotypes in the UK Biobank highlight adipocyte biology

Tanigawa et al.

Supplementary Figures

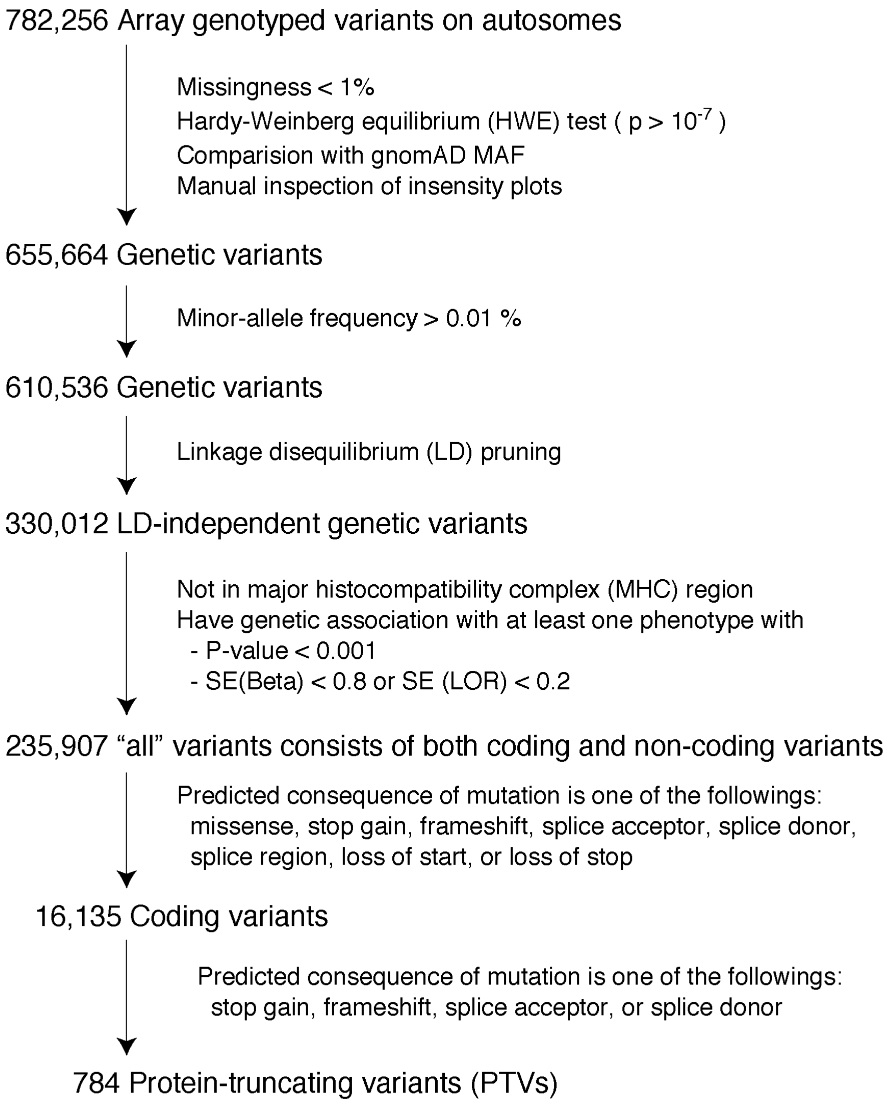

Supplementary Fig. 1. Illustrative summary of the variant filters used in the study. The last three variant sets (“all” variants, coding variants, and PTVs) are used in the study. SE: standard error. LOR: log odds ratio.

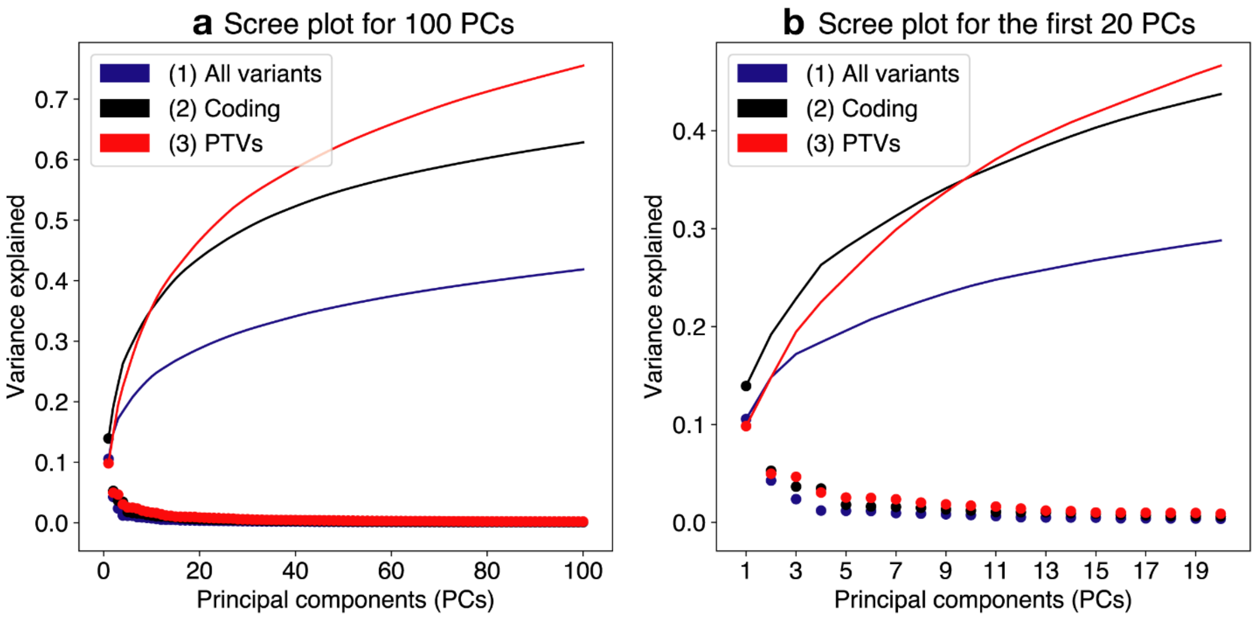

Supplementary Fig. 2. Scree plot from the decomposition of summary statistics. Scree plots summarize variance explained in each of the top 100 (**a**) and 20 (**b**) components. The scree plots are shown for three datasets consists of LD-pruned and QC-filtered sets of array-genotyped variants outside of MHC region: (1) all array-genotyped variants, which includes coding and non-coding variants (blue), (2) coding variants (black), and (3) protein-truncating variants (PTVs, red). For each component, we calculate the variance explained defined as squared eigenvalues divided by the total variance in the original matrix (Methods). We plotted those values as dots and cumulative values as lines.

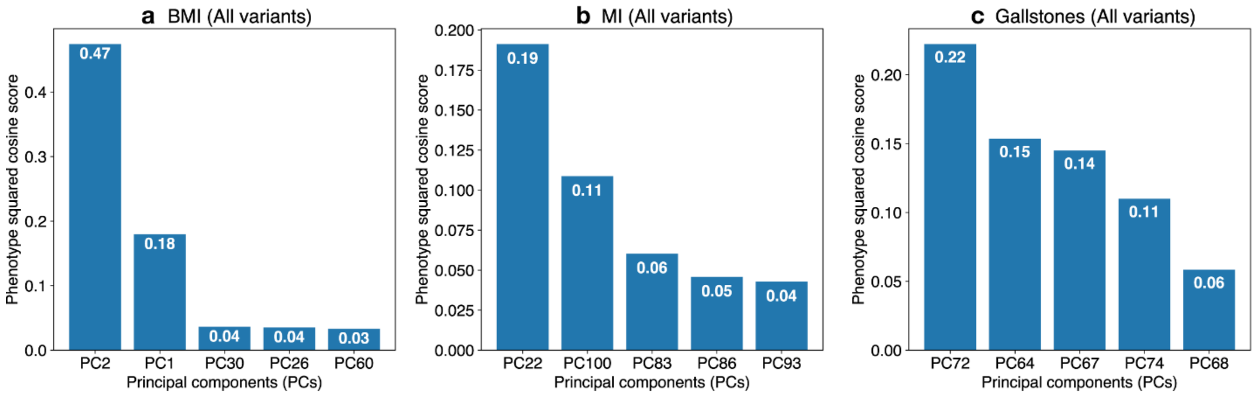

Supplementary Fig. 3. Identification of the key components using "all" dataset. Squared cosine score quantifies relative importance of the key components for a given phenotype. The top five key components are identified for all variant dataset that includes both coding and non-coding variants for three phenotypes: **a** body mass index (BMI), **b** myocardial infarction (MI), and **c** gallstones. The top five key components are shown on the horizontal axis and the corresponding squared cosine scores are shown on the vertical axis.

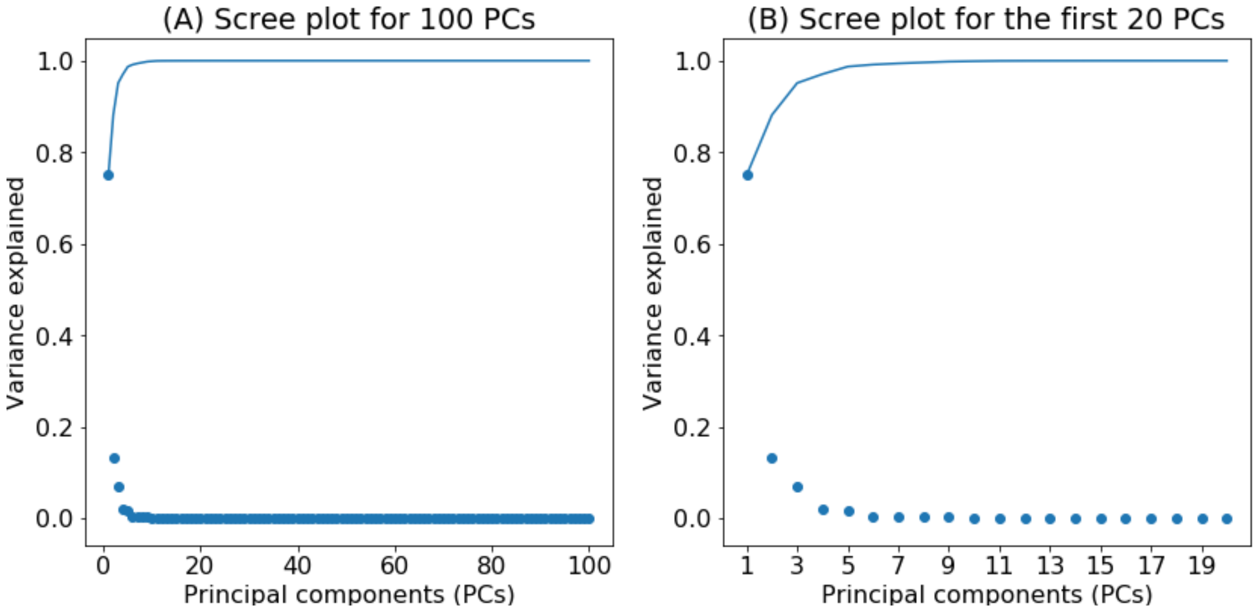

Supplementary Fig. 4. Scree plot from the decomposition of phenotype data. Scree plot summarizes variance explained in the top 100 (**a**) and 20 (**b**) components characterized from the imputed and normalized phenotype data. We calculate the variance explained defined as squared eigenvalues divided by the total variance in the original matrix (Methods). We plotted those values as dots and cumulative values as lines.

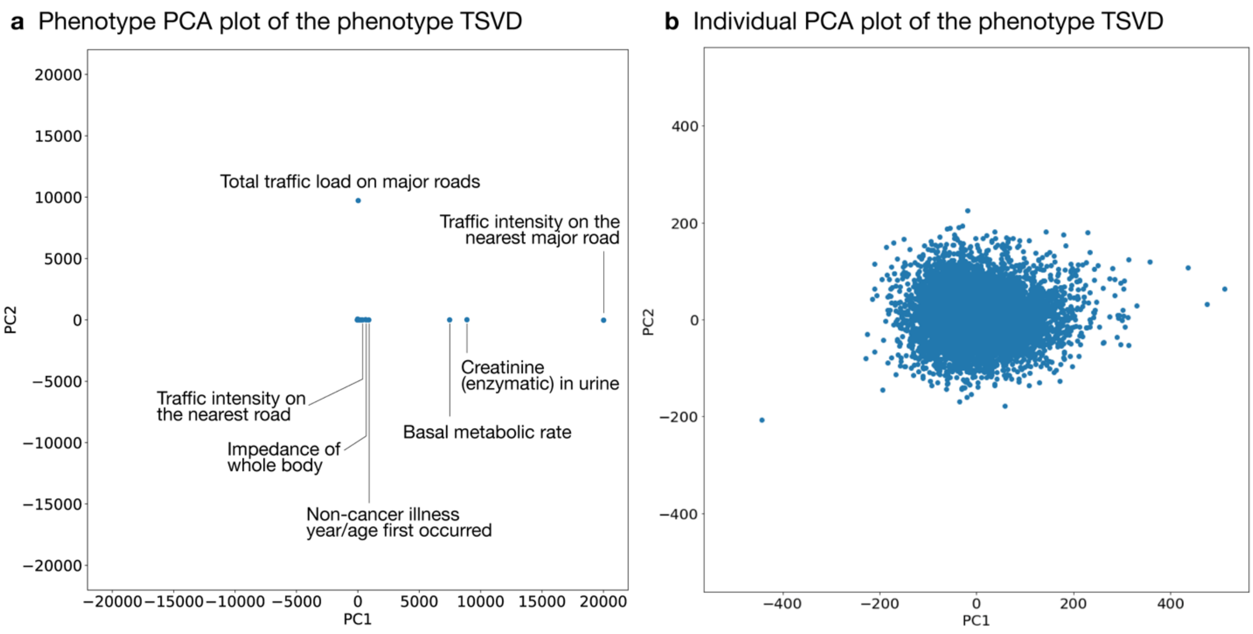

Supplementary Fig. 5. Decomposition of phenotype data. Characterization of latent structures of phenotypic data characterized by truncated singular value decomposition (TSVD) of the imputed and normalized phenotype data. Phenotype (**a**) and Individual (**b**) PCA plots summarizes the first two components.

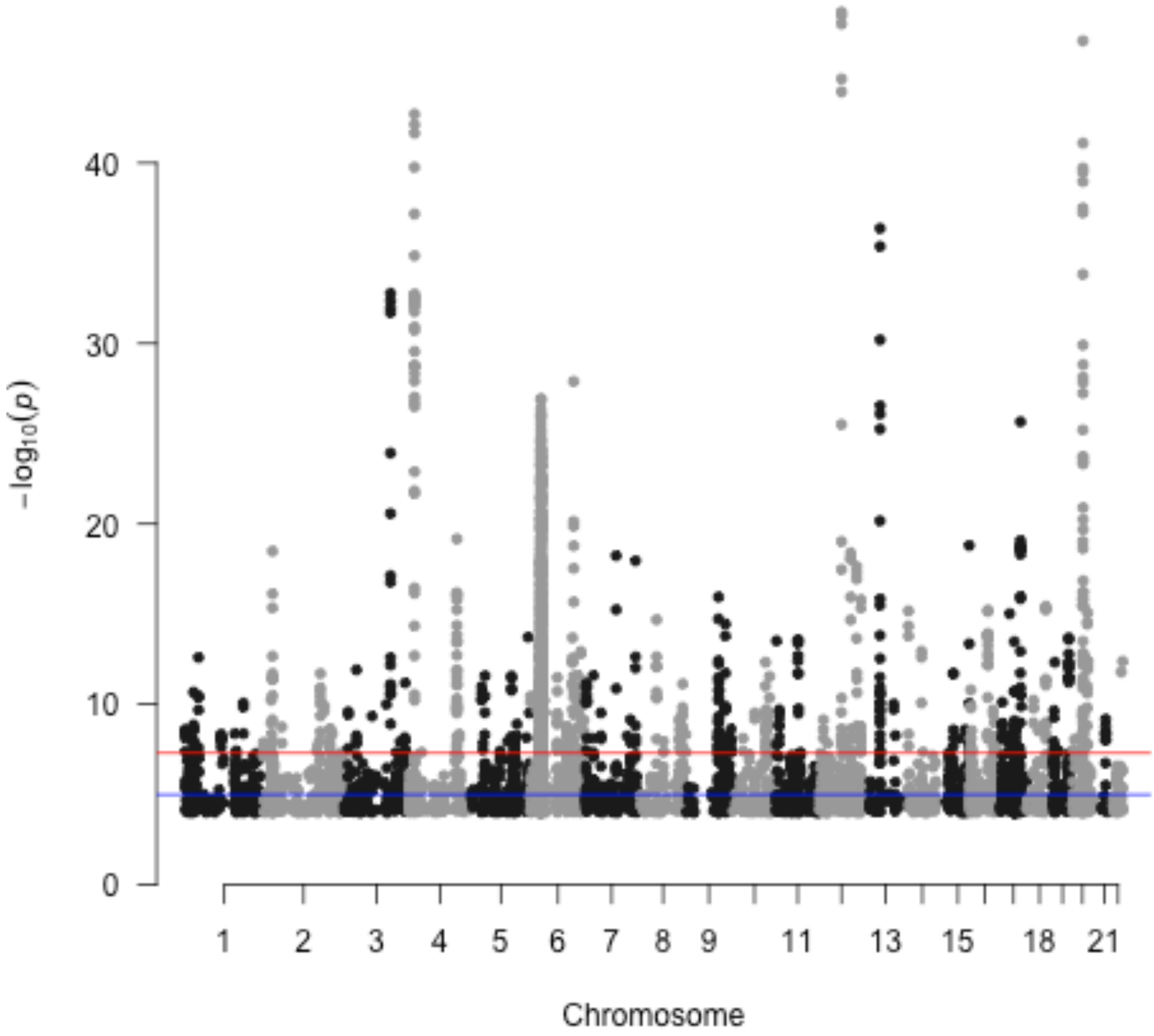

Supplementary Fig. 6. Genome-wide association analysis of the phenotype PCs. After characterizing the phenotype latent space with TSVD on the phenotype data, we performed GWAS analysis. The statistical significance for the first phenotype component is shown in the plot. The variants with $p < 1.0 \times 10^{-4}$ are shown. The red and blue lines indicate genome-wide significance (5.0×10^{-8}) and genome-wide suggestive (5.0×10^{-5}) levels, respectively.

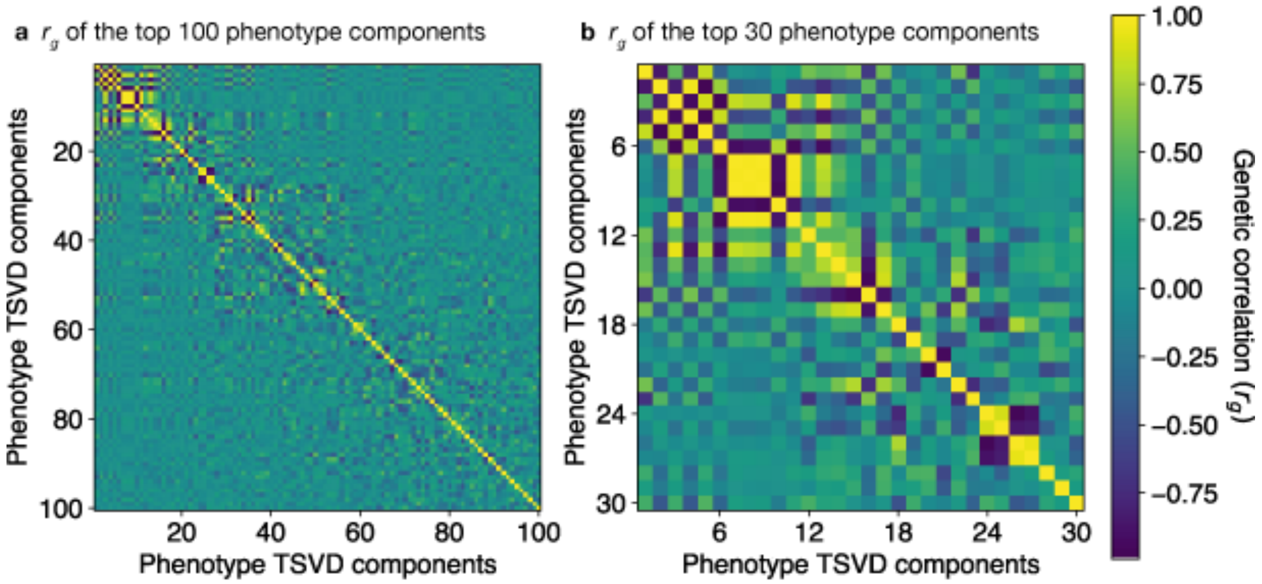

Supplementary Fig. 7. Pairwise similarity of association statistics among phenotype PCs. Genetic correlation (r_g) of phenotype TSVD components shown for the top 100 components (a) and the top 30 components (b), respectively.

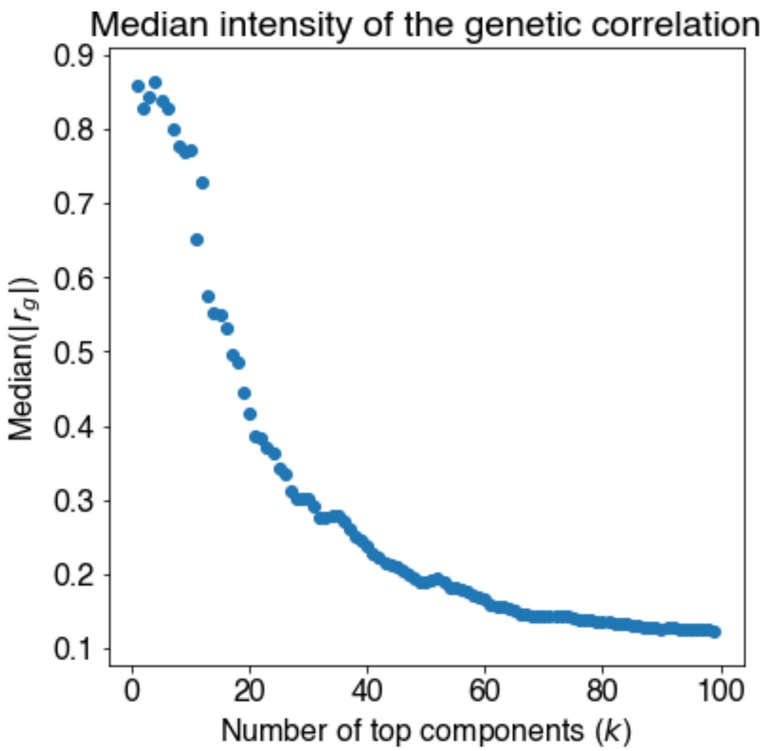

Supplementary Fig. 8. Genetic correlation computed for phenotype PCs. The median of the absolute value of the genetic correlation (r_g) among the top phenotypic components.

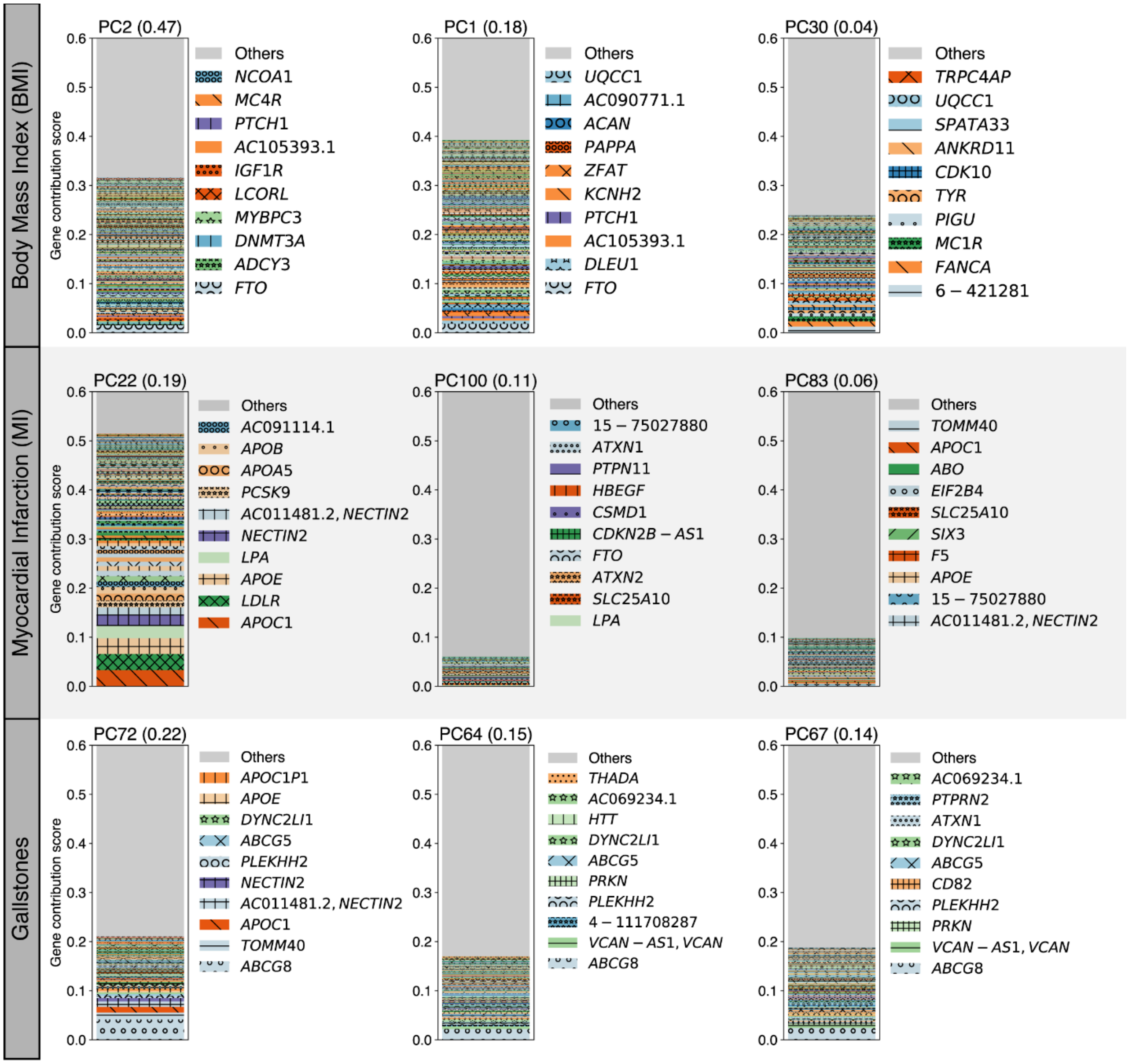

Supplementary Fig. 9. Gene contribution scores for the "all" dataset. Gene contribution scores for the top three key components for body mass index (BMI), myocardial infarction (MI), and gallstones using all variant dataset, which includes both coding and non-coding variants. For each phenotype, the top three key components with their phenotype squared cosine scores are shown on the top of the stacked bar plot and gene contribution scores for each of the components are shown as colored segments. Each colored segment represents a gene with at least 0.05% of contribution scores and the rest of the genes are aggregated as the gray bar at the top. For the visualization, the maximum value of the vertical axis is set to be 0.6. For each component, the labels for the top 10 driving genes are shown. For non-coding variants, we display their genomic coordinates. Source data are provided in Supplementary Data 2.

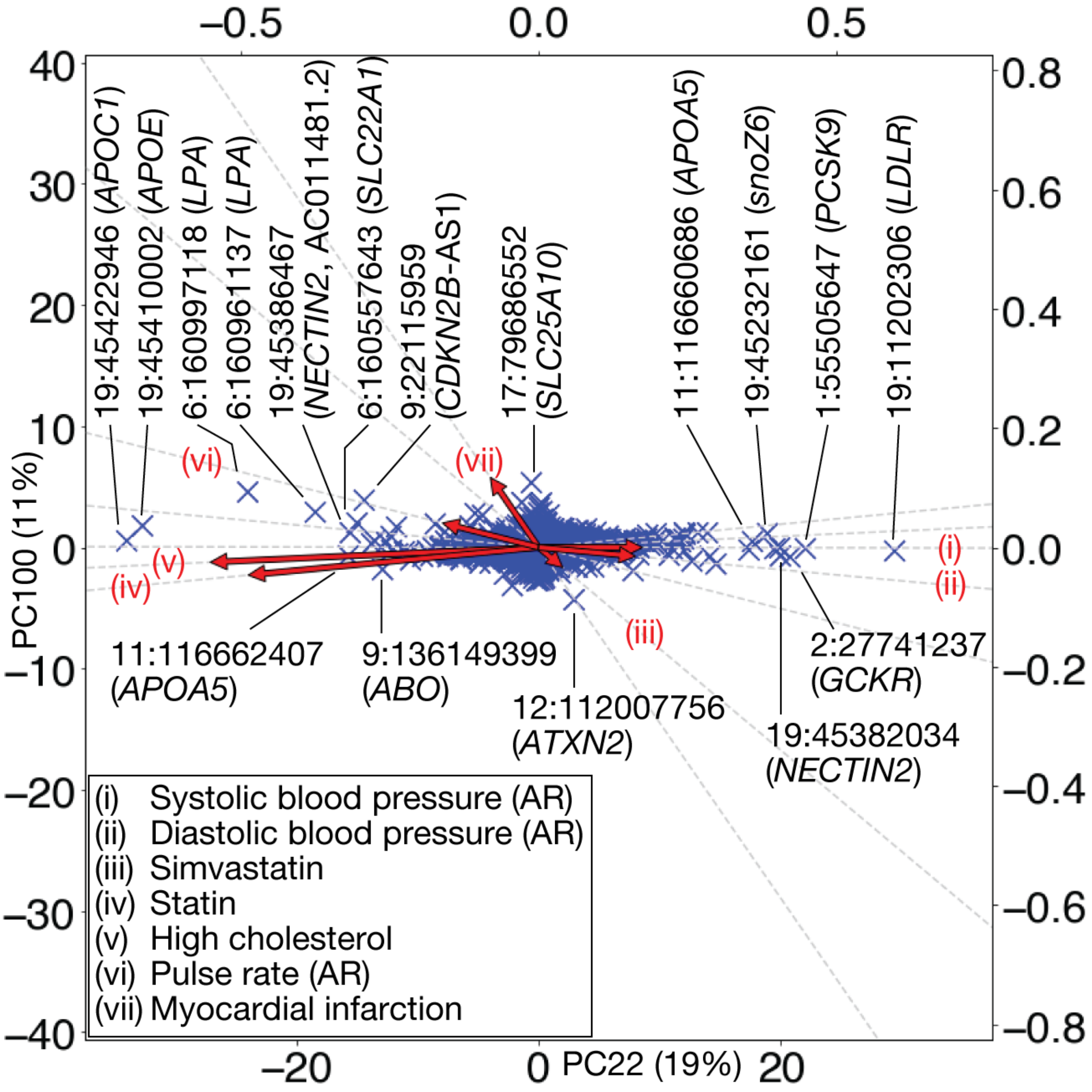

Supplementary Fig. 10. The biplot annotation of the top two components for MI. Variant PCA plot with biplot annotation for the top two key components for myocardial infarction using "all" dataset. Genetic variants projected into the top two key components, PC22 (horizontal axis) and PC100 (vertical axis) are shown as scatter plot. Variants are annotated with gene symbols. Directions of genetic associations for relevant phenotypes are annotated as red arrows using the secondary axes (Methods). Abbreviations. AR: automated reading.

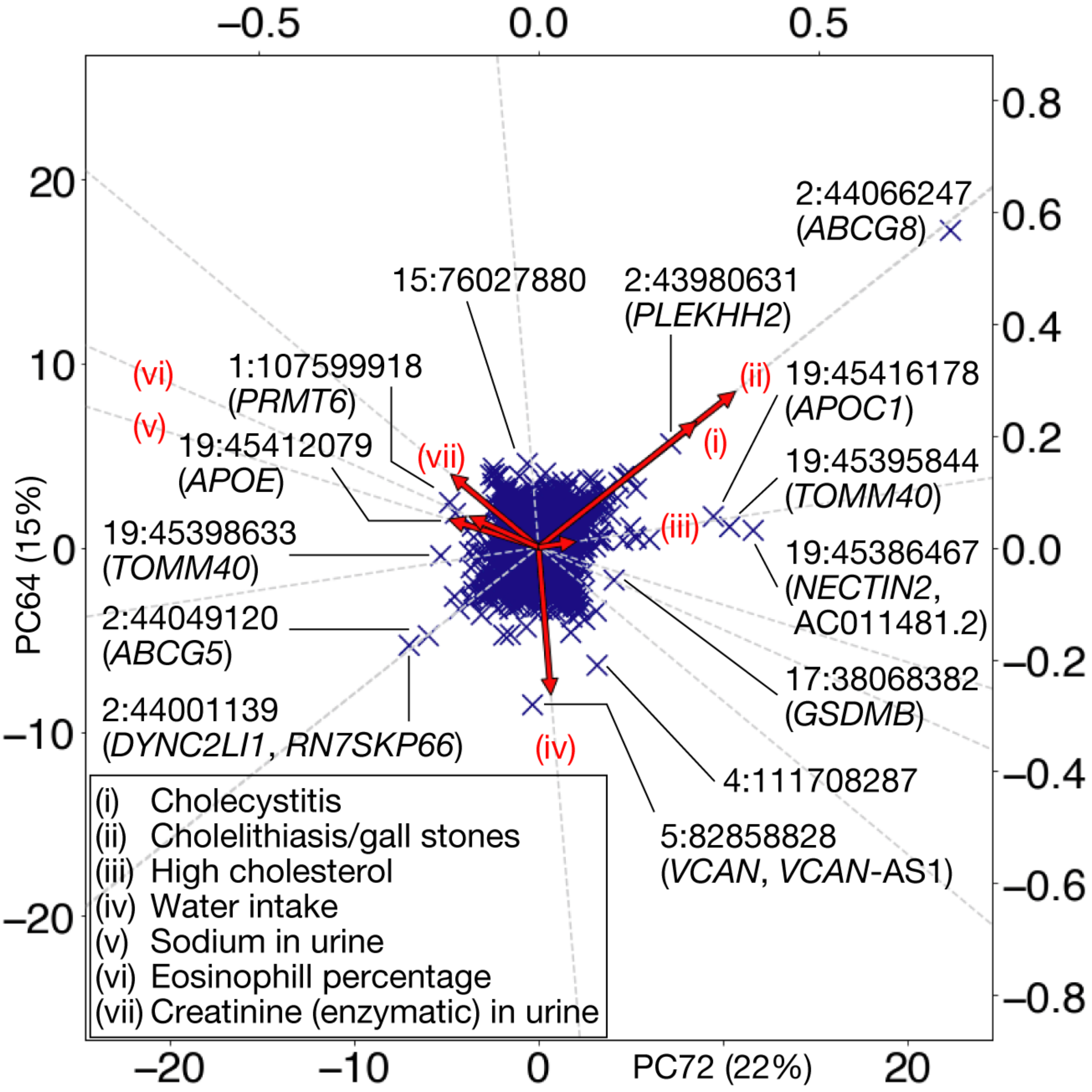

Supplementary Fig. 11. The biplot annotation of the top two components for gallstones. Variant PCA plot with biplot annotation for the top two key components for gallstones using "all" dataset. Genetic variants projected into the top two key components, PC72 (horizontal axis) and PC64 (vertical axis). Variants are annotated with gene symbols. Directions of genetic associations for relevant phenotypes are annotated as red arrows using the secondary axes (Methods).

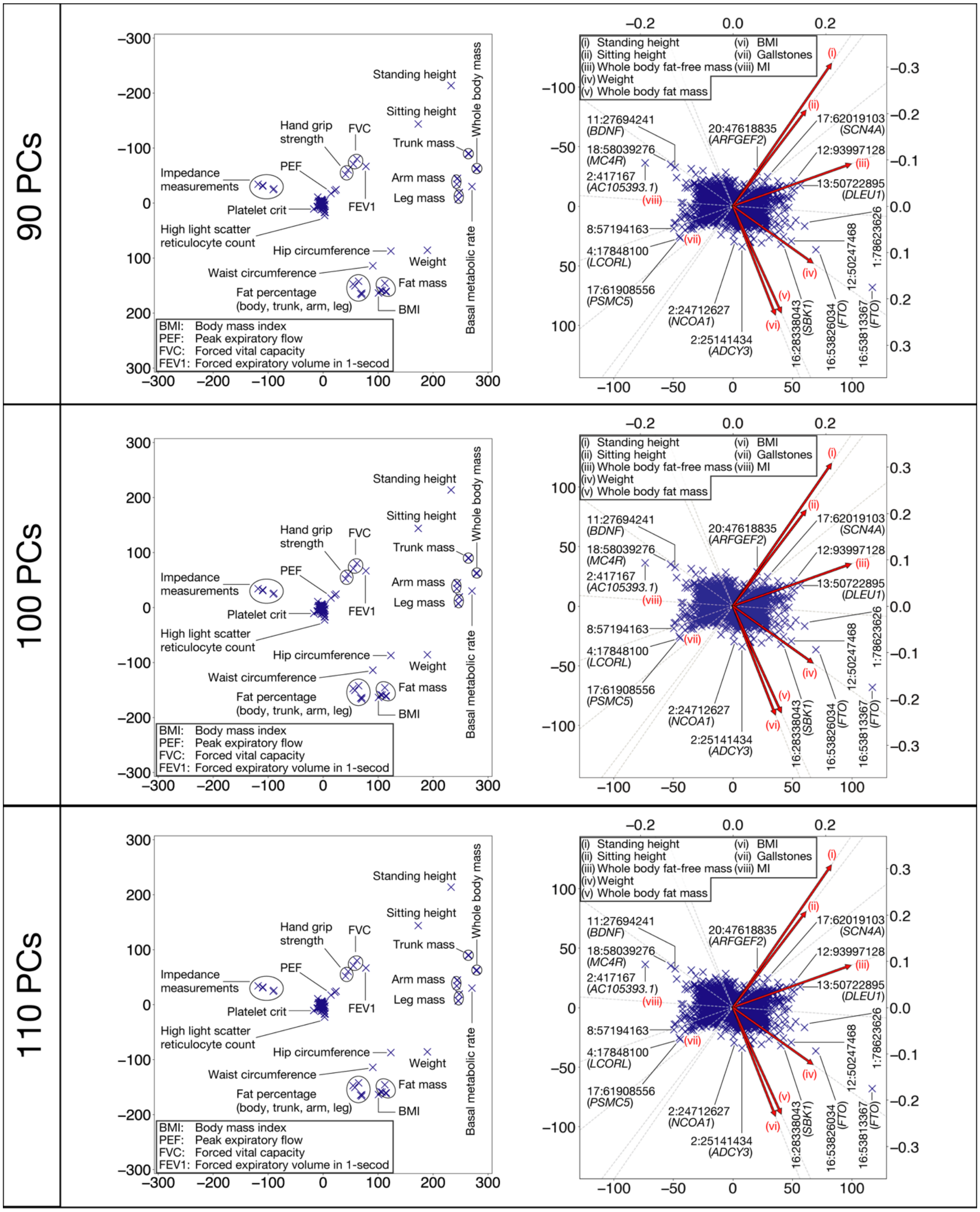

Supplementary Fig. 12. Robustness analysis of the top two DeGAs components. Comparison of the top two DeGAs components by robustness analysis with respect to the number of latent factors in DeGAs. The phenotype PCA plot (left) and the variant PCA plot with the biplot annotations (right) are shown (Methods). To cope with the sign indeterminacy of the latent components, the direction of PC2 is reversed in the plots for TSVD with 90 PCs.

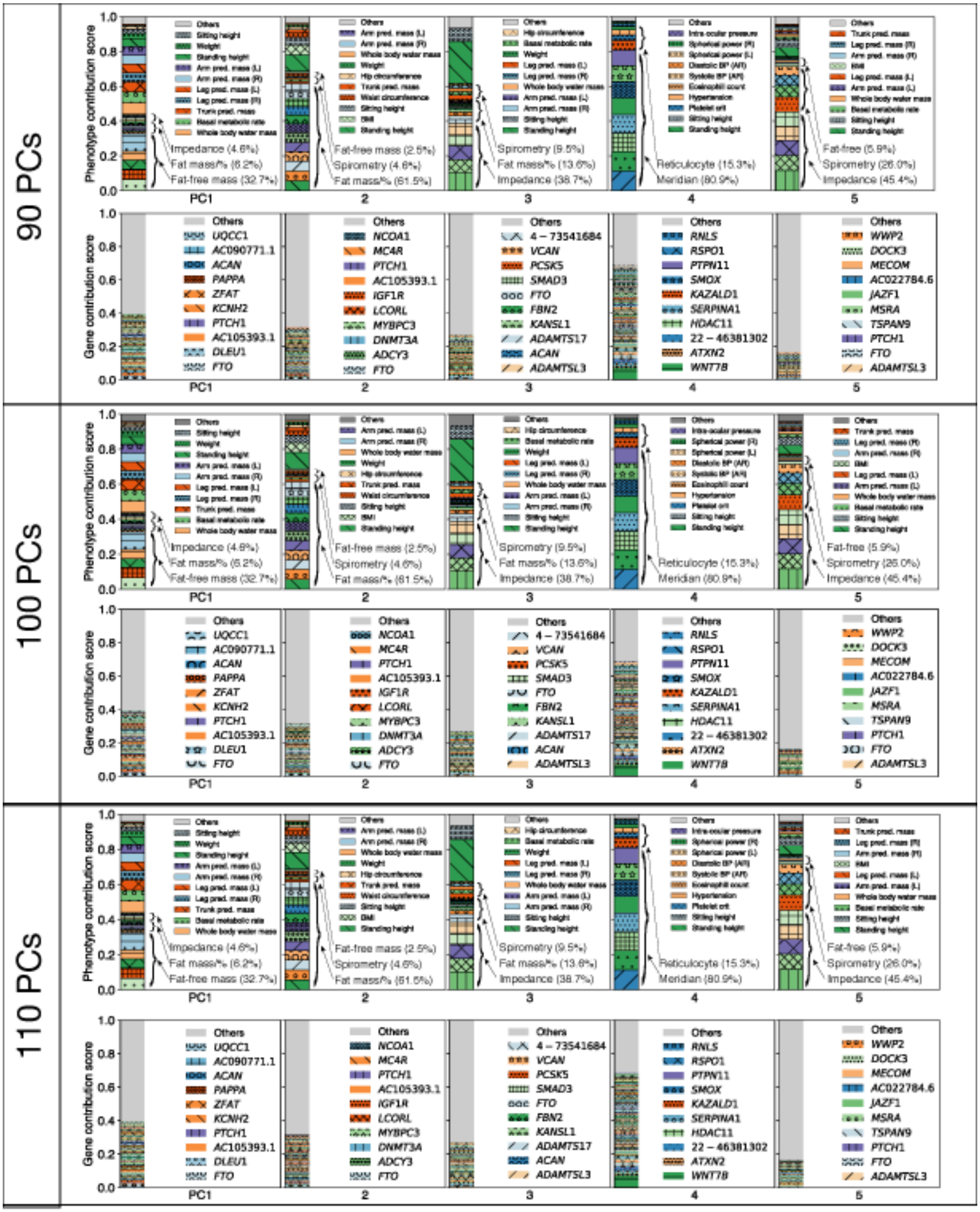

Supplementary Fig. 13. Robustness analysis of the top five DeGAs components. Comparison of the top five DeGAs components by robustness analysis with respect to the number of latent factors in DeGAs. The phenotype and gene contribution scores are shown. Each colored segment represents a phenotype or gene with at least 0.5% and 0.05% of phenotype and

gene contribution scores, respectively, and the rest is aggregated as others on the top of the stacked bar plots. The major contributing phenotype groups (Methods, Supplementary Table 2) and additional top 10 phenotypes and the top 10 genes for each component are annotated.

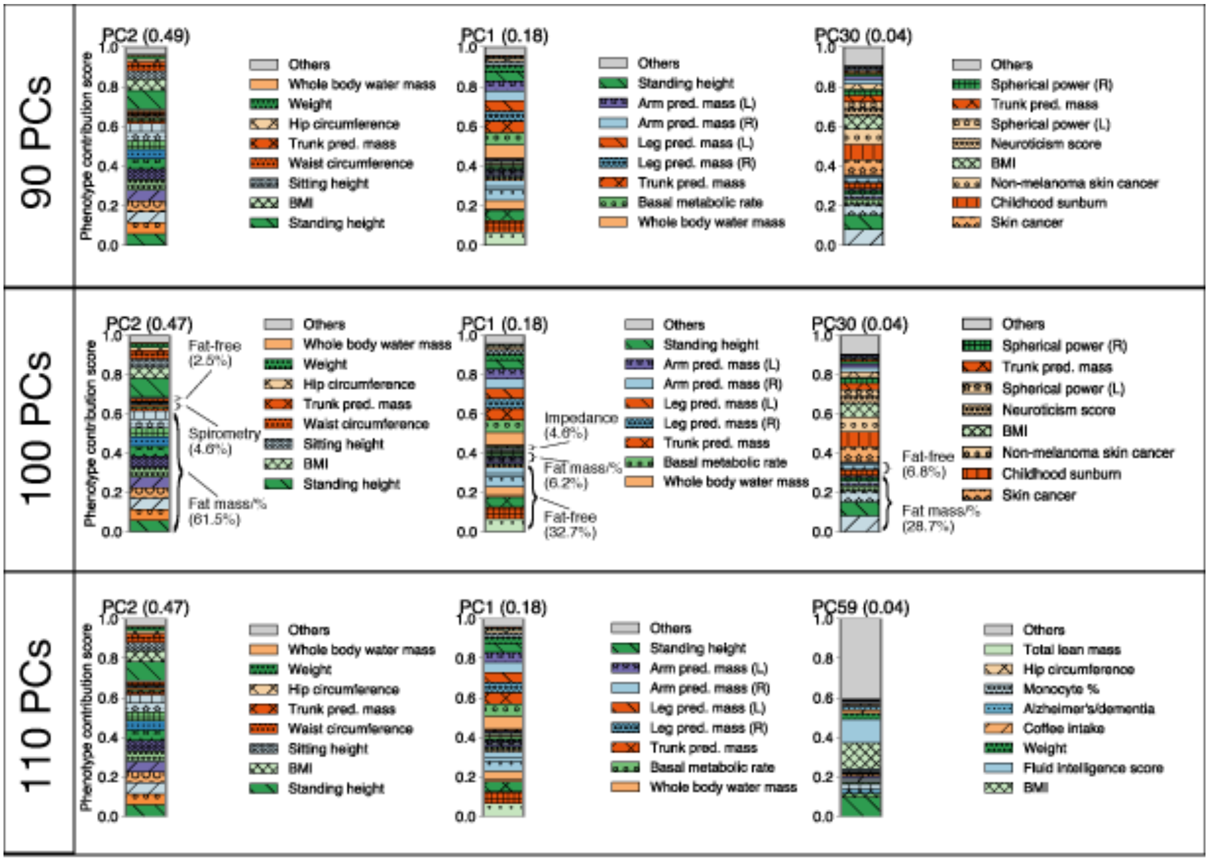

Supplementary Fig. 14. Robustness analysis of the top three components for BMI. Comparison of the key components for body mass index (BMI) by robustness analysis with respect to the number of latent factors in DeGAs. For each condition, the top three key components with their phenotype squared cosine scores are shown on the top of the stacked bar plot and phenotype contribution scores for each of the components are shown as colored segments. Each colored segment represents a gene with at least 0.5% of contribution scores and the rest of the phenotypes are aggregated as the gray bar at the top. For each component, the labels for the top 6 driving phenotypes are shown.

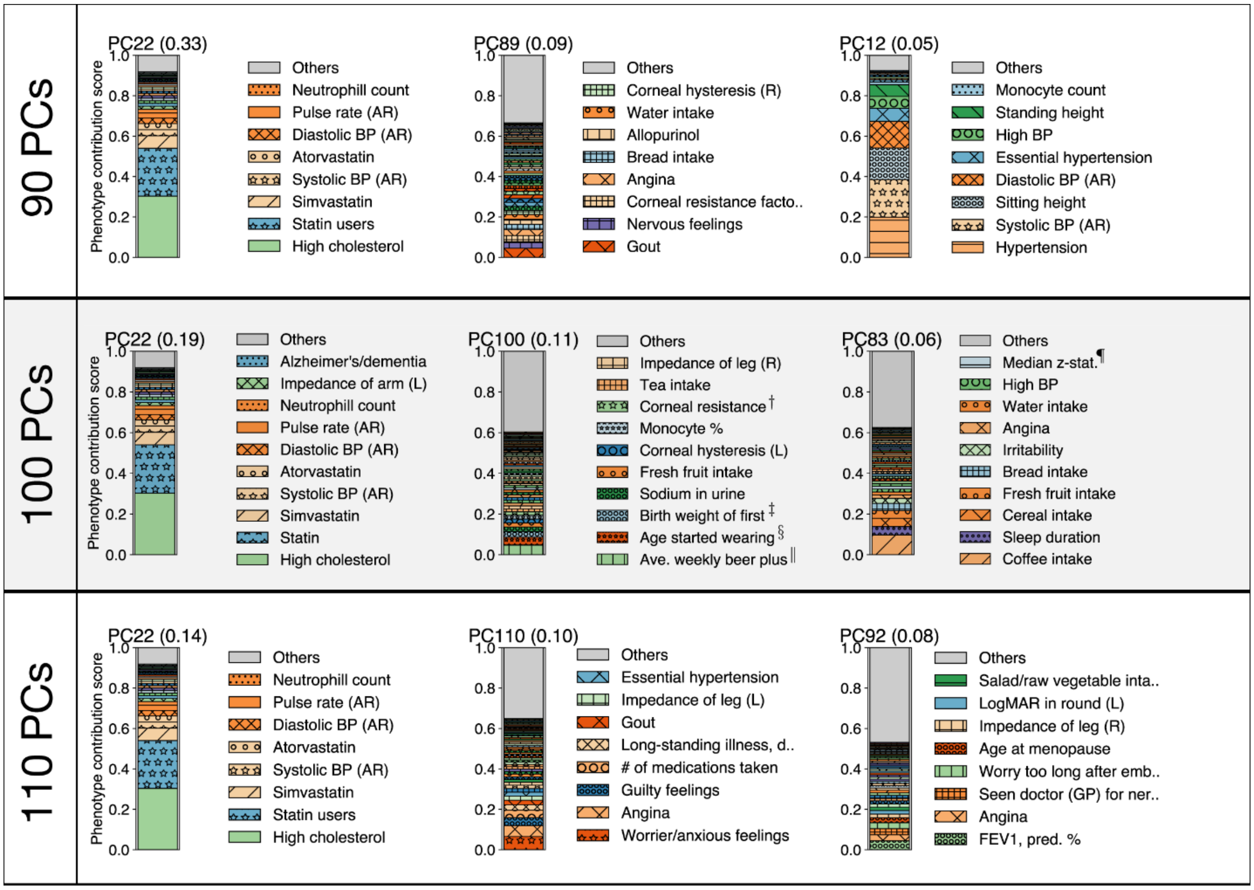

Supplementary Fig. 15. Robustness analysis of the top three components for MI. Comparison of the key components for myocardial infarction (MI) by robustness analysis with respect to the number of latent factors in DeGAs. For each condition, the top three key components with their phenotype squared cosine scores are shown on the top of the stacked bar plot and phenotype contribution scores for each of the components are shown as colored segments. Each colored segment represents a gene with at least 0.5% of contribution scores and the rest of the phenotypes are aggregated as the gray bar at the top. For each component, the labels for the top 6 driving phenotypes are shown.

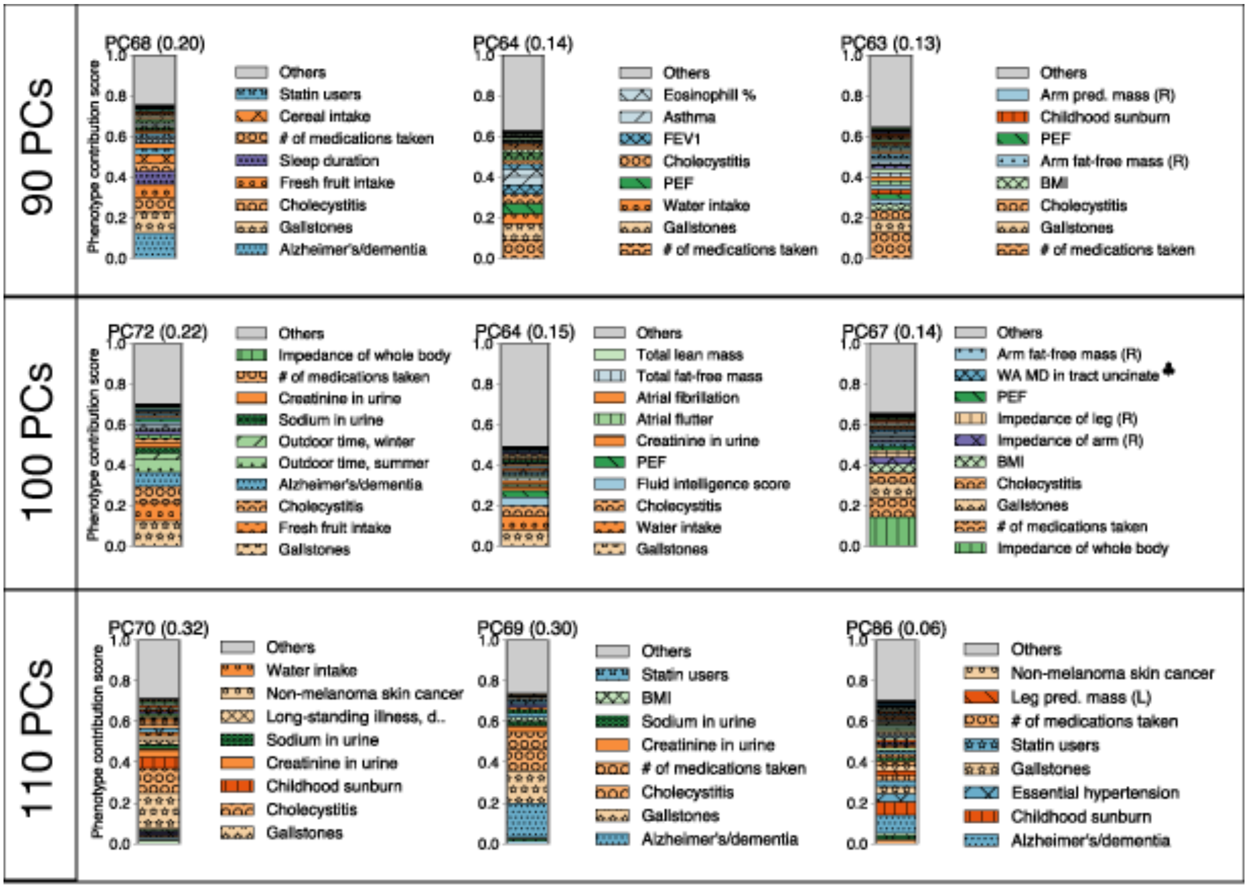

Supplementary Fig. 16. Robustness analysis of the top three components for gallstones. Comparison of the key components for gallstones by robustness analysis with respect to the number of latent factors in DeGAs. For each condition, the top three key components with their phenotype squared cosine scores are shown on the top of the stacked bar plot and phenotype contribution scores for each of the components are shown as colored segments. Each colored segment represents a gene with at least 0.5% of contribution scores and the rest of the phenotypes are aggregated as the gray bar at the top. For each component, the labels for the top 6 driving phenotypes are shown.

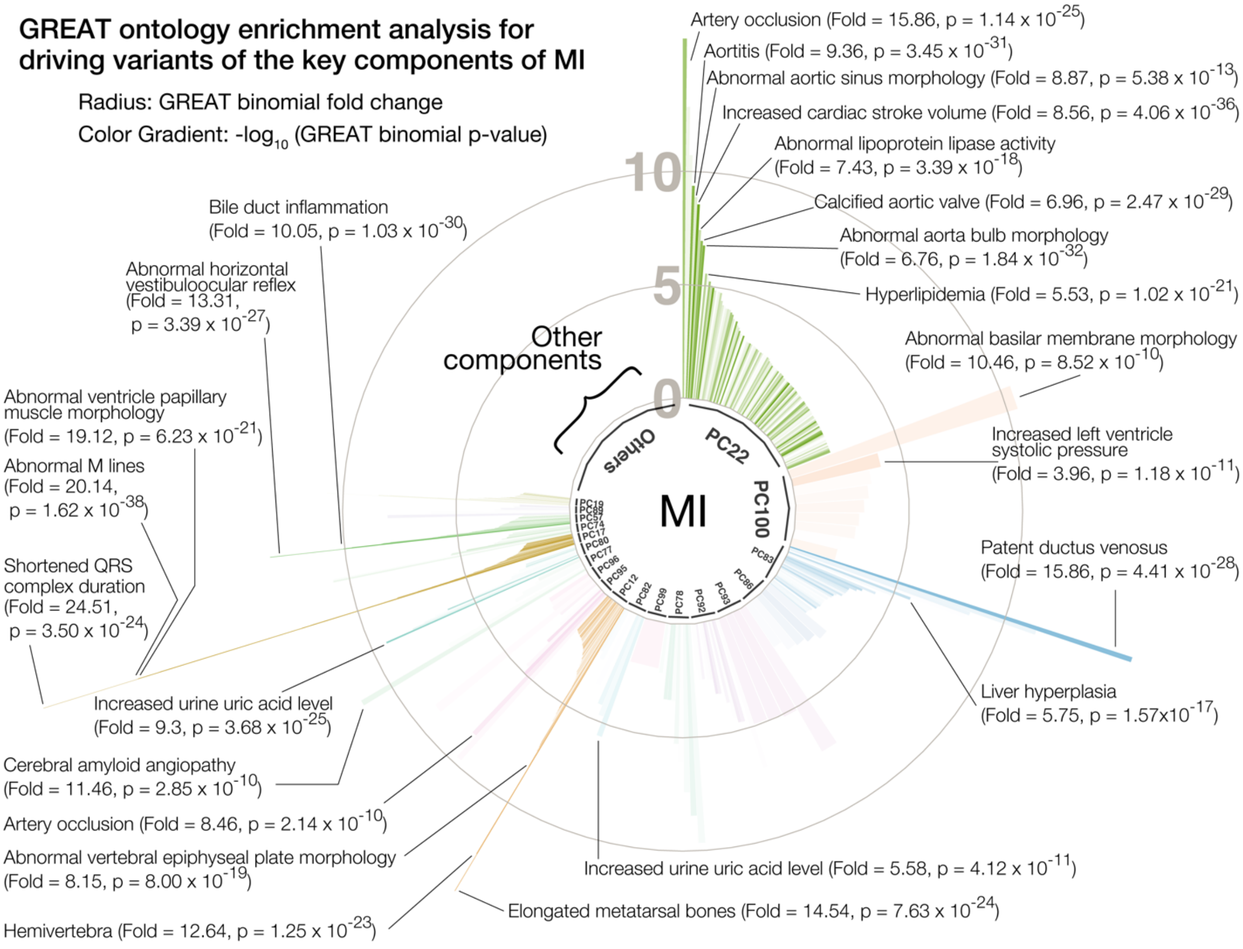

Supplementary Fig. 17. The GREAT enrichment analysis for MI. Biological characterization of driving non-coding and coding variants of the key components for myocardial infarction (MI) with the genomic region enrichment analysis tool (GREAT) using the all variants dataset. The key components are shown proportional to their squared cosine score along with significantly enriched terms in mouse genome informatics (MGI) phenotype ontology. The radius represents binomial fold change and the color gradient represents -log$_{10}$ (p-value) from GREAT ontology enrichment analysis. Source data are provided in Supplementary Data 4.

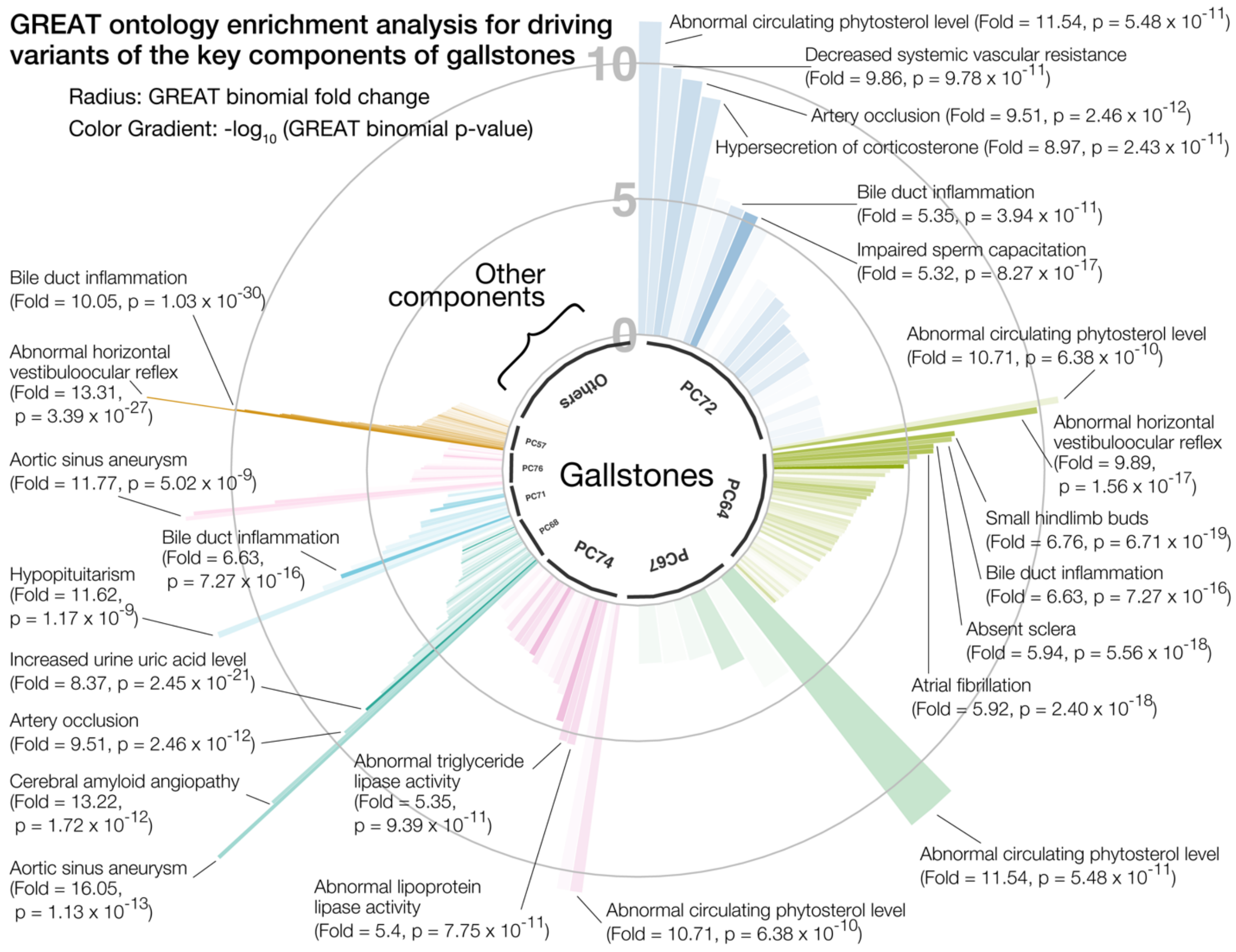

Supplementary Fig. 18. The GREAT enrichment analysis for gallstones. Biological characterization of driving non-coding and coding variants of the key components for gallstones with the genomic region enrichment analysis tool (GREAT) using the all variants dataset. The key components are shown proportional to their squared cosine score along with significantly enriched terms in mouse genome informatics (MGI) phenotype ontology. The radius represents binomial fold change and the color gradient represents -log$_{10}$(p-value) from GREAT ontology enrichment analysis. Source data are provided in Supplementary Data 5.

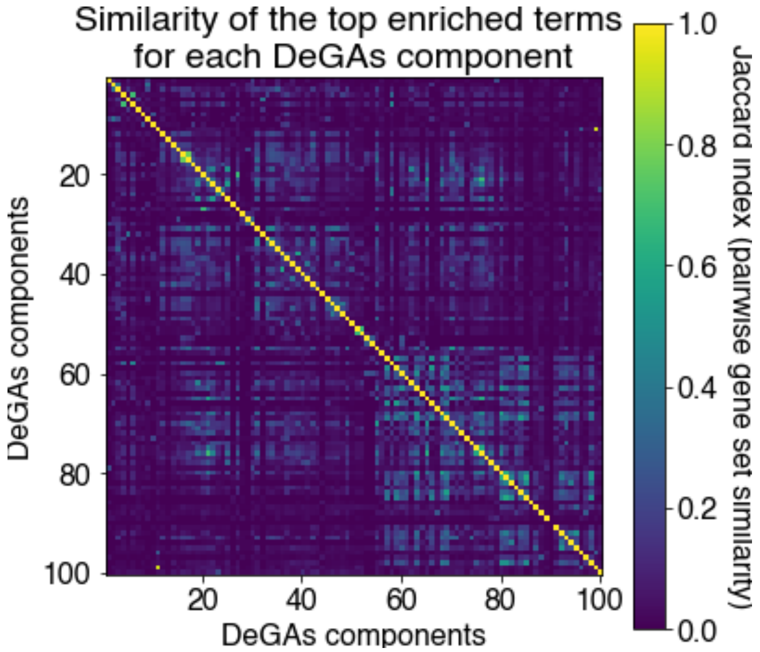

Supplementary Fig. 19. Similarity of the top enriched terms for each DeGAs component. For each DeGAs component, we took the top enriched ontology terms identified by GREAT and obtained the list of genes annotated with that term. Using these gene sets, we quantified the pairwise gene set similarity across the 100 DeGAs components using a set similarity measure, Jaccard Index, that ranges from 0 (completely disjoint) to 1 (completely identical) (Methods).

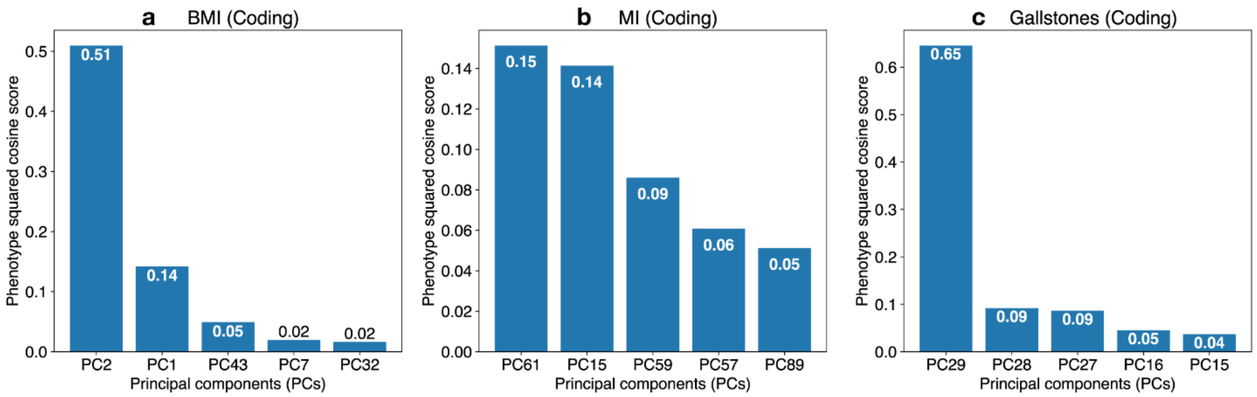

Supplementary Fig. 20. Identification of the key components using coding dataset. Identification of the key components with phenotype squared cosine scores using coding dataset. Squared cosine score quantifies relative importance of the key components for a given phenotype. The top five key components are identified for coding dataset for three phenotypes: **a** body mass index (BMI), **b** myocardial infarction (MI), and **c** gallstones. The top five key components are shown on the horizontal axis and the corresponding squared cosine scores are shown on the vertical axis.

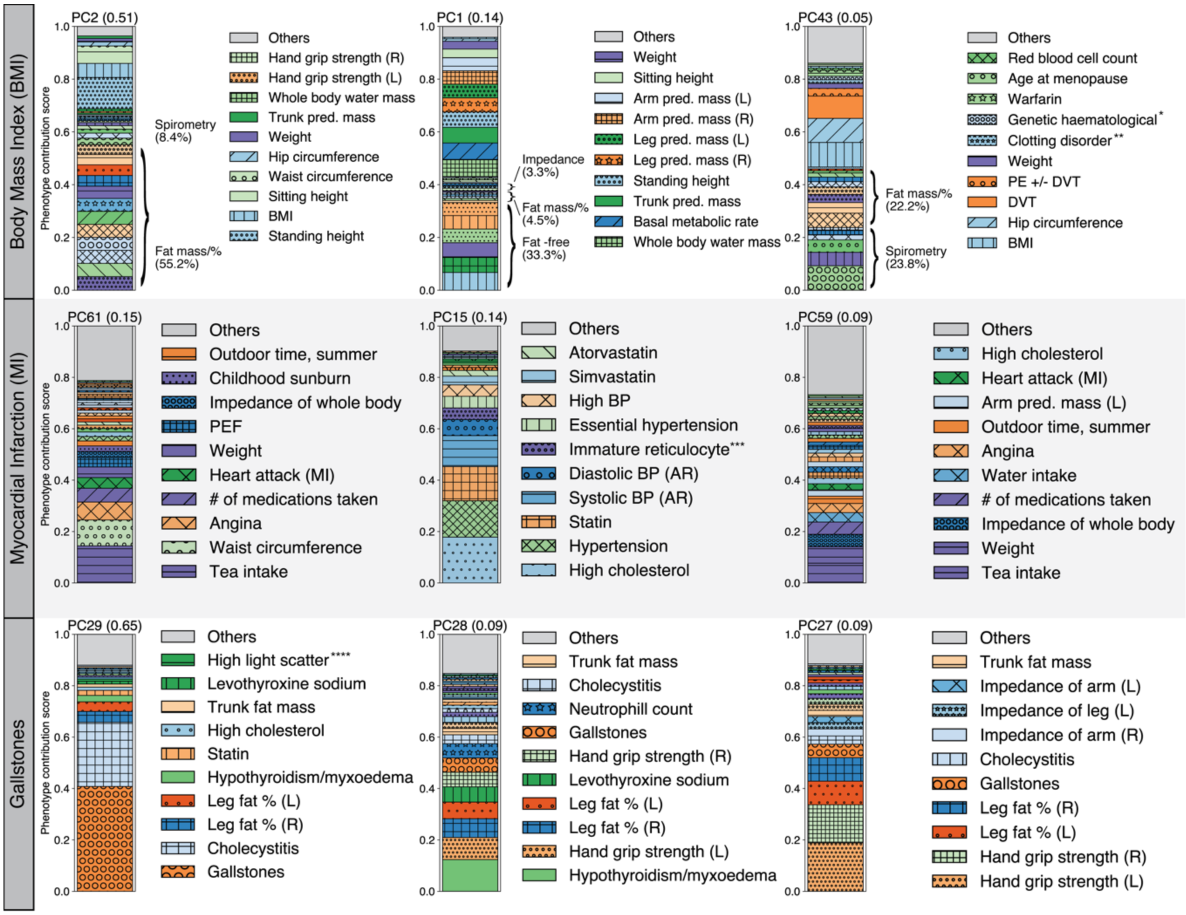

Supplementary Fig. 21. Phenotype contribution scores for the coding dataset. Phenotype contribution scores for the top three key components for body mass index (BMI), myocardial infarction (MI), and gallstones using coding dataset. For each phenotype, the top three key components with their phenotype squared cosine scores are shown on the top of the stacked bar plot and phenotype contribution scores for each of the components are shown as colored segments. Each colored segment represents a phenotype with at least 0.5% of contribution scores and the rest of the genes are aggregated as the gray bar at the top. For BMI, additional phenotype grouping is applied (Methods, Supplementary Table 2). For each component, the labels for the top 10 driving genes are shown. Source data are provided in Supplementary Data 2.

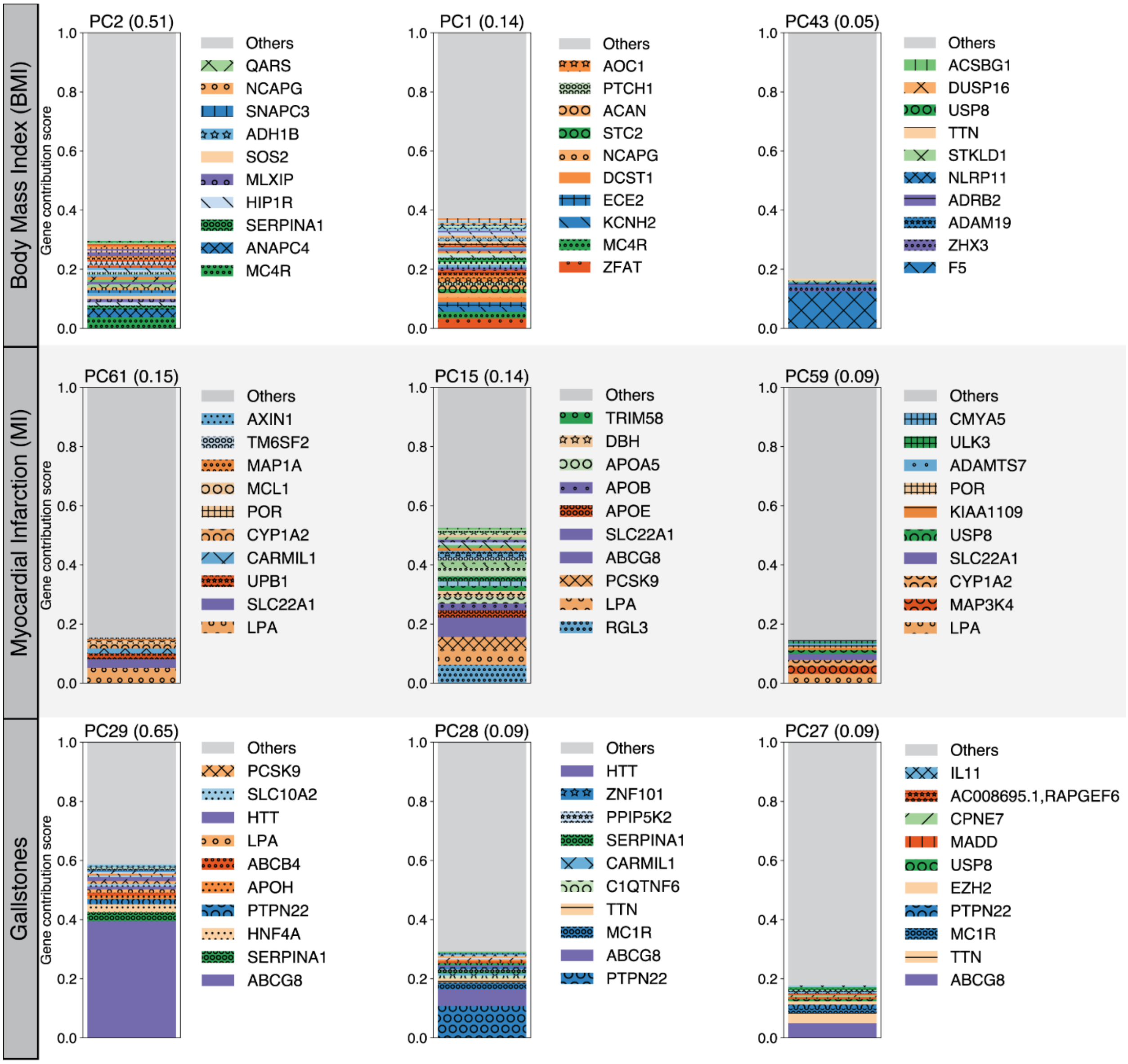

Supplementary Fig. 22. Gene contribution scores for the coding dataset. Gene contribution scores for the top three key components for body mass index (BMI), myocardial infarction (MI), and gallstones using coding dataset. For each phenotype, the top three key components with their phenotype squared cosine scores are shown on the top of the stacked bar plot and gene contribution scores for each of the components are shown as colored segments. Each colored segment represents a gene with at least 0.05% of contribution scores and the rest of the genes are aggregated as the gray bar at the top. For each component, the labels for the top 10 driving genes are shown. Source data are provided in Supplementary Data 2.

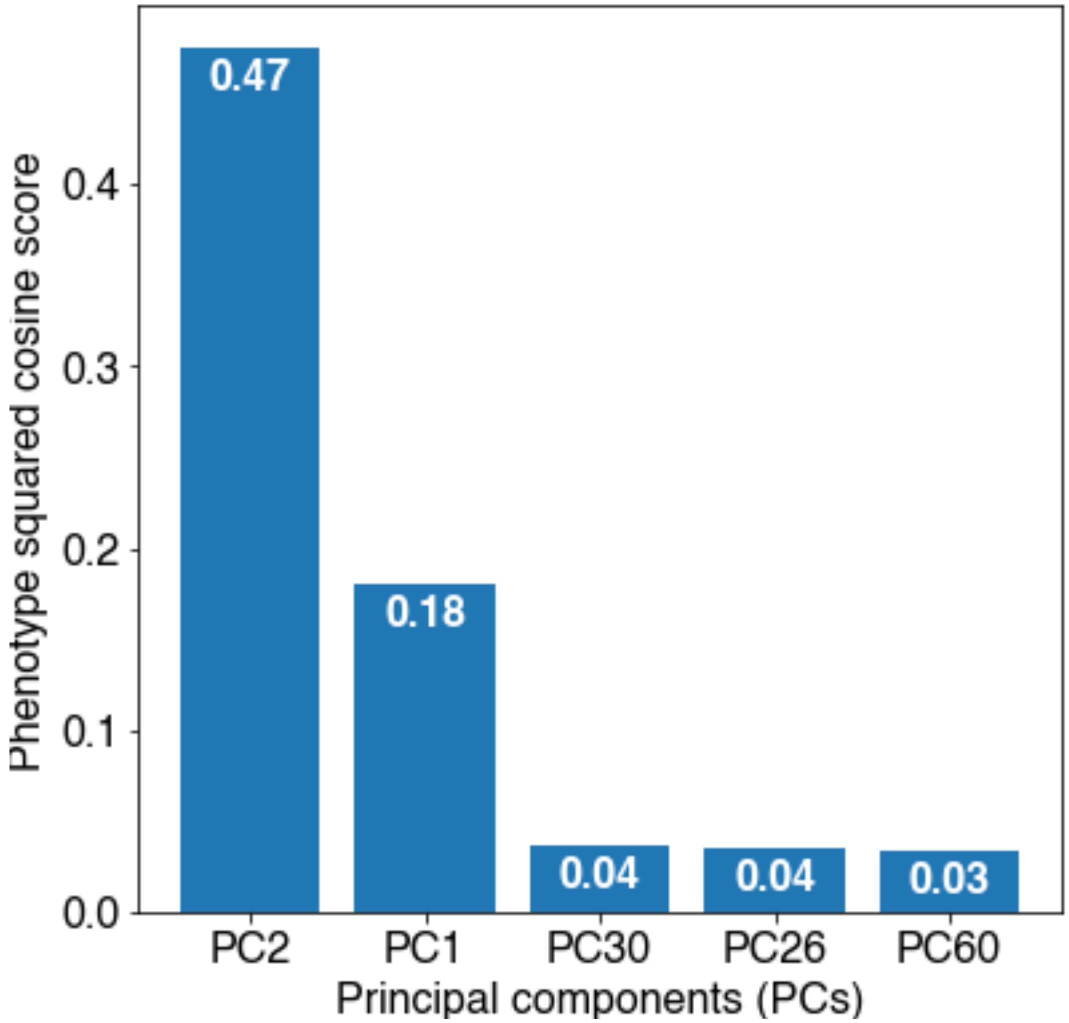

Supplementary Fig. 23. Identification of the key components using PTVs dataset. Identification of the key components for BMI with phenotype squared cosine scores using the PTVs dataset. The top five key components are shown on the horizontal axis and the corresponding squared cosine scores are shown on the vertical axis.

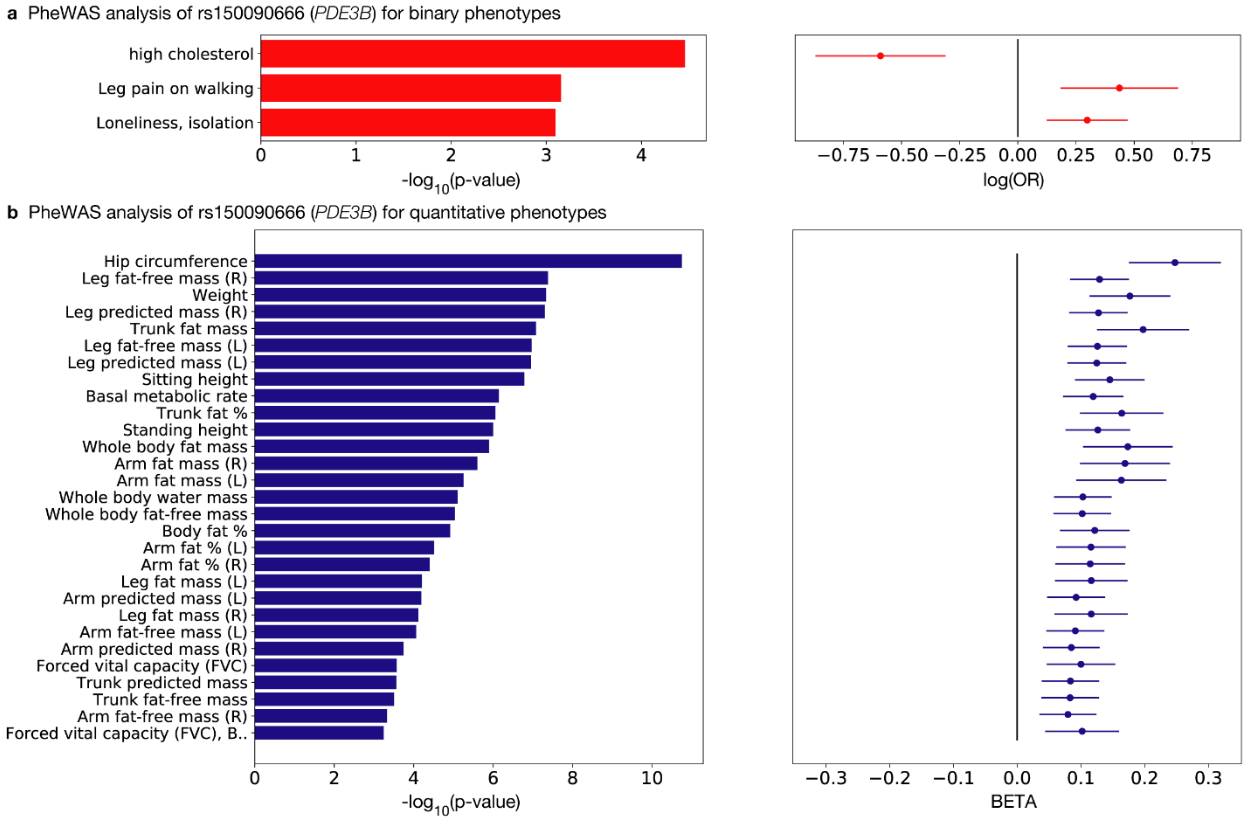

Supplementary Fig. 24. PheWAS scan for rs150090666, a stop-gain variant in *PDE3B*. The p-values (left) and log odds ratio (binary phenotypes, shown as red) or beta (quantitative phenotypes, shown as blue) (right) along with 95% confidence interval are shown for the phenotypes with minimum case count of 1,000 (binary phenotypes, **a**) or 1,000 individuals with non-missing values (quantitative phenotypes, **b**) and strong association ($p \leq 0.001$) and with this variants among all the phenotypes used in the study (n = 337,199 White British individuals in the UK Biobank for binary traits and n > 255,000 for each quantitative trait, Supplementary Table 3). Source data are provided in Supplementary Table 4.

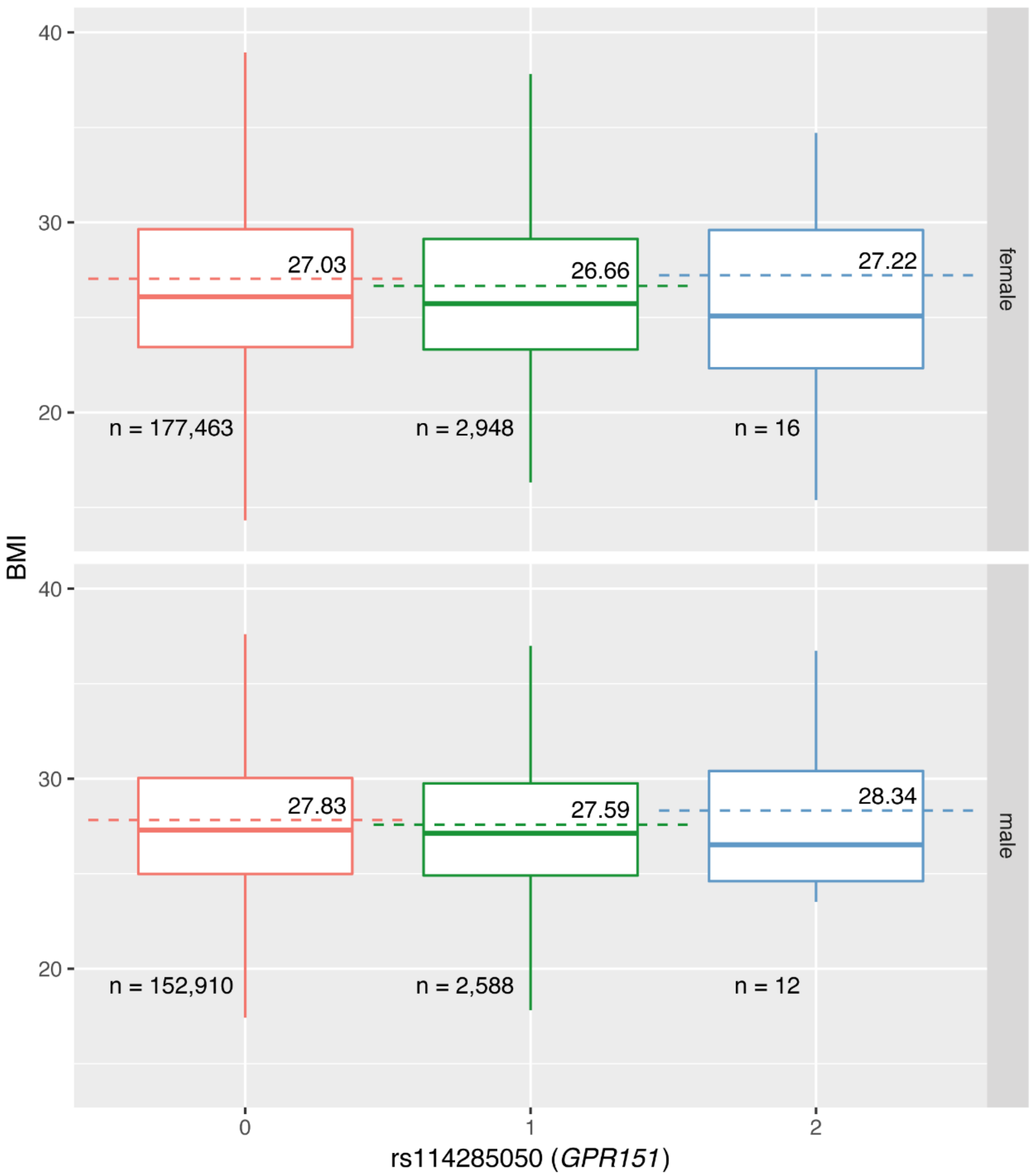

Supplementary Fig. 25. The distribution of BMI stratified by sex and rs114285050. The phenotype values are stratified by genotype of rs114285050, a stop-gain variant in *GPR151*. The outliers are removed from the plot and the mean values are annotated and shown as dashed lines. In the box plots, the median, two hinges (the first and the third quartiles) and two whiskers are shown. The upper whisker extends from the hinge to the largest value no further than 1.5 * IQR from the hinge (where IQR is the inter-quartile range, or distance between the first and third quartiles). The number of carriers of the variant (n) is shown at the bottom.

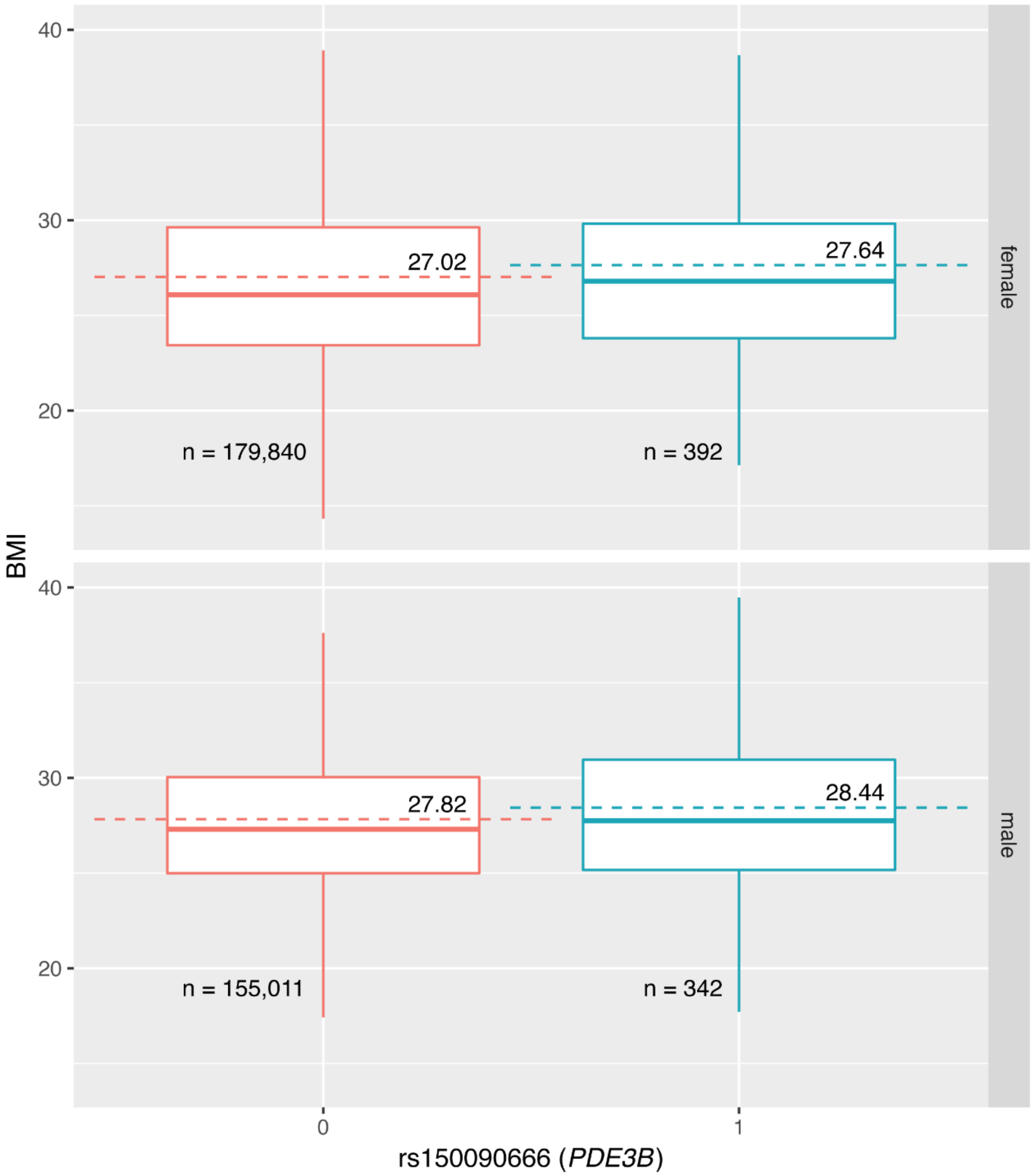

Supplementary Fig. 26. The distribution of BMI stratified by sex and rs150090666. The phenotype values are stratified by genotype of rs150090666, a stop-gain variant in *PDE3B*. The outliers are removed from the plot and the mean values are annotated and shown as dashed lines. In the box plots, the median, two hinges (the first and the third quartiles) and two whiskers are shown. The upper whisker extends from the hinge to the largest value no further than 1.5 * IQR from the hinge (where IQR is the inter-quartile range, or distance between the first and third quartiles). The number of carriers of the variant (n) is shown at the bottom.

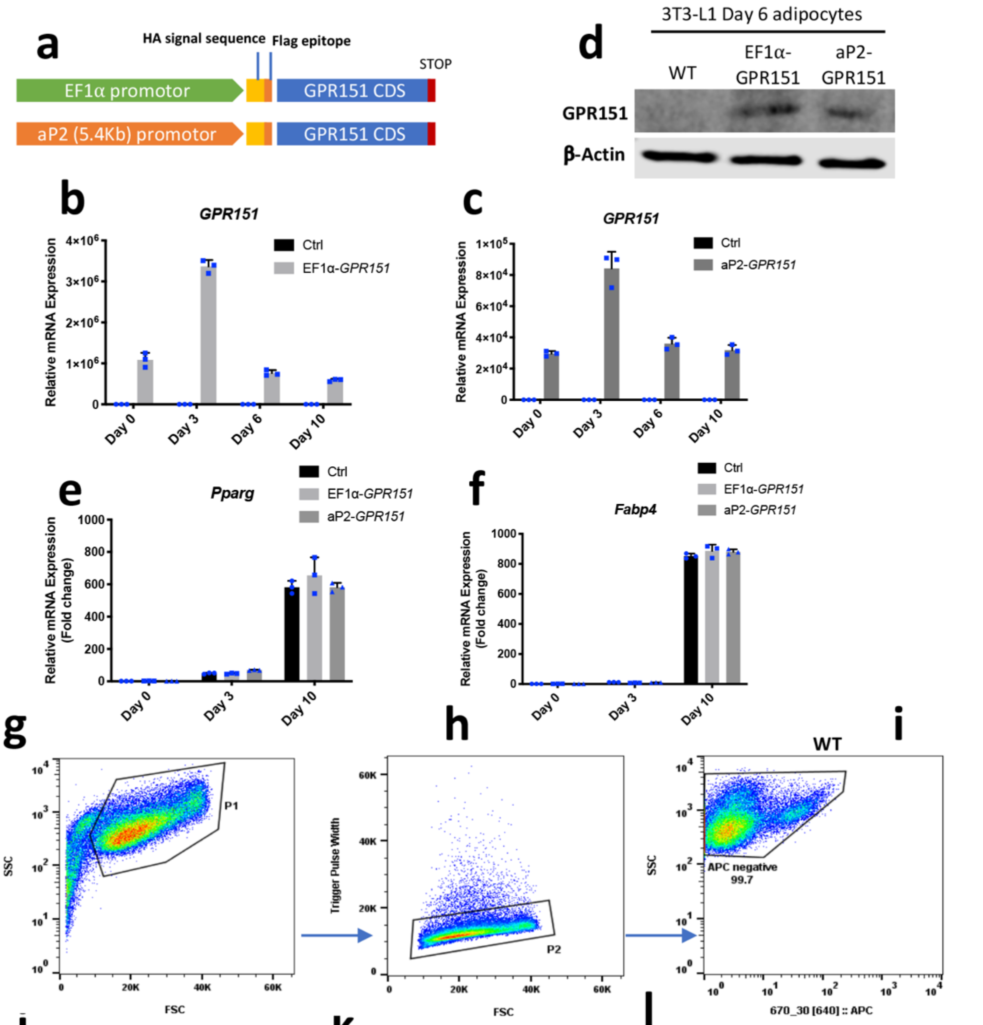

l

Sample Name	Subset Name	Freq. of Total	Freq. of Parent
WT	Ungated	100	100
	P1	80.9	80.9
	P2	78.1	96.5
	APC negative	75.9	99.7
EF1α-*GPR151*	Ungated	100	100
	P1	73.5	73.5
	P2	68.6	93.4
	APC negative	64.6	94.2
	APC positive	1.55	2.25
aP2-*GPR151*	Ungated	100	100
	P1	68.8	68.8
	P2	62.6	91.1
	APC negative	58.3	93.1
	APC positive	0.35	0.55

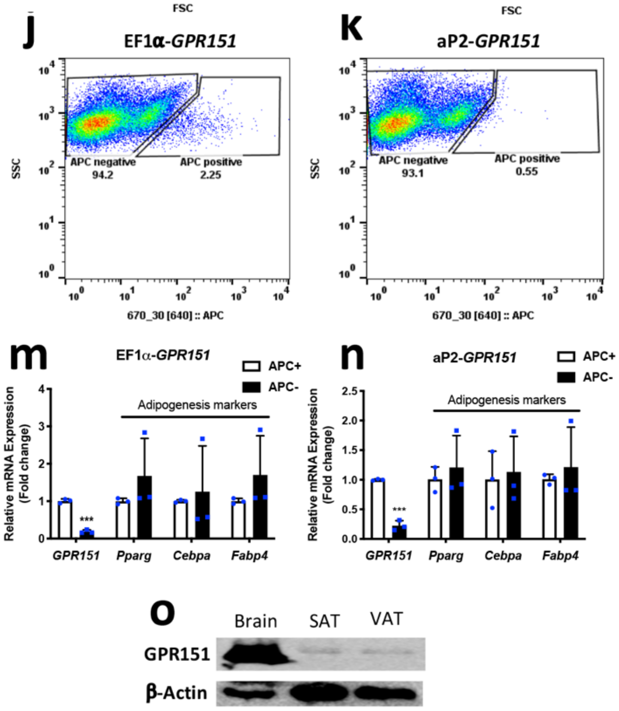

Supplementary Fig. 27. Effects of *GPR151* overexpression on 3T3-L1 adipogenesis. a Structure of *GPR151* overexpression construct driven by either EF1α or aP2 promotor. **b-d** Confirmation of *GPR151* overexpression at both mRNA (**b-c**) and protein levels (**d**) in 3T3-L1 cells during adipogenesis. n=3 independent experiments. **e-f** qPCR analysis of the effect of *GPR151* overexpression on adipogenesis markers, *Pparg* (**e**) and *Fabp4* (**f**). n=3 independent experiments. **g-i** FACS gating strategy to sort APC+ and APC- adipocytes used for qPCR analysis of *GPR151* and adipogenesis markers presented in Supplementary Fig. 27m-n. Cells were initially selected by size, on the basis of forward scatter (FSC) and side scatter (SSC) (**g**). Cells were then gated on both FSC and SSC singlets to ensure that individual cells were analyzed (**h**). Non-infected Day 6 3T3-L1 wild-type (WT) adipocytes were used to determine background fluorescence levels (**i**). **j-l** Representative FACS collection gates used to sort Day 6 3T3-L1 adipocytes infected with either EF1α-*GPR151* (**j**) or aP2-*GPR151* (**k**) (shown as APC positive), in comparison to WT (shown as APC negative). The abundance of the relevant cell population in post-sort fractions were listed in **l**. **m-n** Relative mRNA levels of *GPR151* and adipogenic markers (*Pparg, Cebpa, Fabp4*) in purified APC+ and APC- cells from Day 6 3T3-L1 adipocytes infected by either EF1α-*GPR151* (**m**) or aP2-*GPR151* (**n**). n=3 independent experiments. **o** Comparison of protein levels of GPR151 in mouse brain, subcutaneous adipose tissue (SAT) and visceral adipose tissue (VAT). For bar plots, means ± SEM are shown. ND: not-detectable. Source data are provided as a Source Data file.

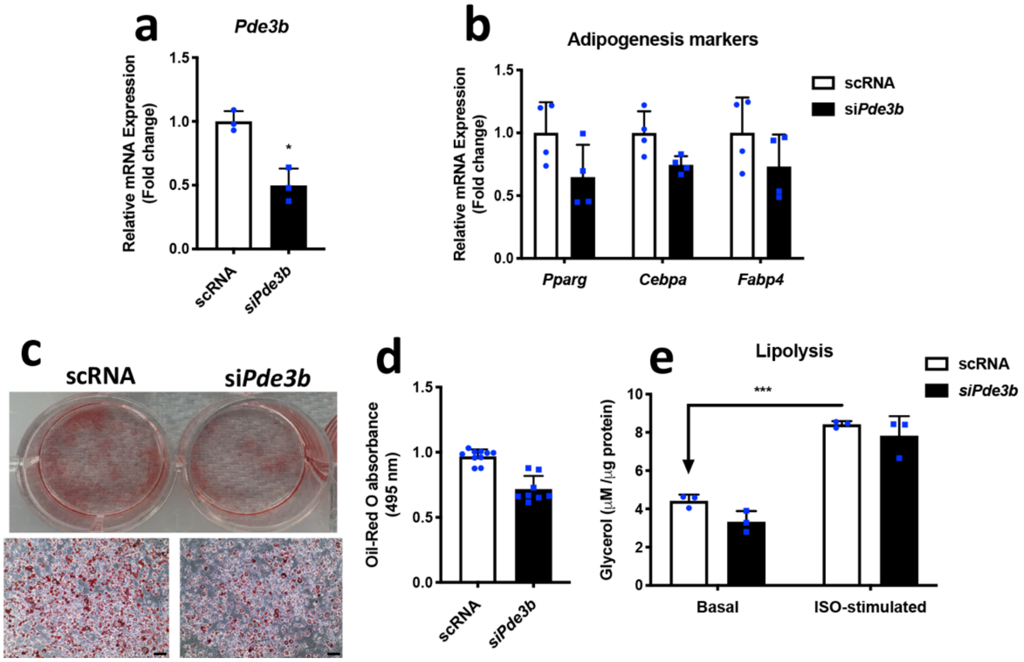

Supplementary Fig. 28. Effects of *Pde3b* knockdown in 3T3-L1 adipogenesis. a qPCR analysis of *Pde3b* mRNA knockdown in 3T3-L1 preadipocytes. n=3 independent experiments. **b** qPCR analysis of the effect of si*Pde3b* knockdown on adipogenesis markers, *Pparg*, *Cebpa* and *Fabp4*. n=4 independent experiments. **c-d** Oil-Red O staining (**c**) and quantification (**d**) of lipid droplets in scRNA- or si*Pde3b*-tansfected adipocytes. 10x magnification. Scale bar = 100μm. n=10 independent culture wells for scRNA, n=8 for si*Pde3b*. **e** lipolysis assays of scRNA- or si*Pde3b*-tansfected adipocytes. n=3 independent experiments. Means ± SEM are shown (***p-value<0.001, *p-value<0.05). scRNA: scrambled siRNA. ISO: isoproterenol. Source data are provided as a Source Data file.

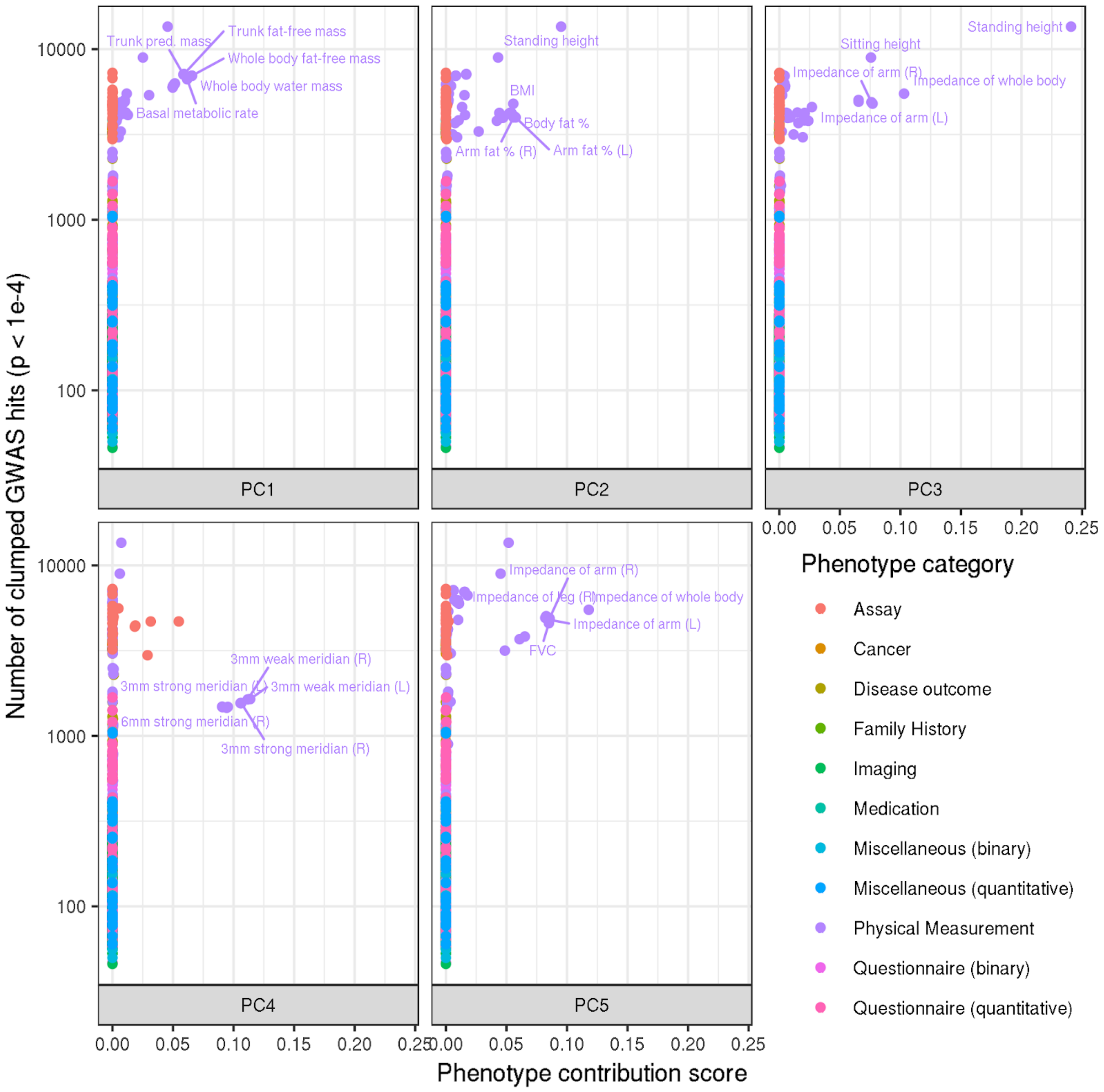

Supplementary Fig. 29. Phenotype contribution scores and the number of GWAS hits. The phenotype contribution score (x-axis) and the number of clumped GWAS hits ($p < 1e-4$, y-axis) are shown for the first five DeGAs components (PC1-5). Each point is a phenotype and they are grouped and colored by phenotype categories defined in Supplementary Table 1 and Supplementary Data 1.

Supplementary Tables

Supplementary Table 1. List of phenotype categories used in our study with examples. The type column indicates whether the phenotype is binary (B) or quantitative (Q). Number of phenotypes, example phenotype for each, and data source are shown. L: described in a previously published literature[1], F: the UK Biobank data field ID, and C: the UK Biobank data category ID.

Phenotype category	Type	Number of phenotypes	Example	Data source
Disease outcome	B	363	Hypertension	L
Cancer	B	46	Skin cancer	L
Family History	B	10	High blood pressure	L
Medication	B	709	Aspirin intake	F: 20003
Questionnaire (binary)	Q	49	Wears glasses or contact lenses	C:100025
Imaging	Q	683	Volume of white matter	C:100003
Physical Measurement	Q	122	Standing height	C:100006
Assay	Q	34	Red blood cell (erythrocyte) count	C:100079
Questionnaire (quantitative)	Q	62	Sleep duration	C:100079
Miscellaneous (binary)	B	19	Ever attempted suicide	
Miscellaneous (quantitative)	Q	41	Number of medications taken	

Supplementary Table 2. Phenotype groupings for visualization. The list of phenotype groups used in the phenotype contribution score plots are summarized.

Phenotype groups	List of phenotypes in the group
Fat-free	Arm fat-free mass (left)
	Arm fat-free mass (right)
	Leg fat-free mass (left)
	Leg fat-free mass (right)
	Total fat-free mass
	Trunk fat-free mass
	Whole body fat-free mass
Fat	Android fat mass
	Android tissue fat percentage
	Arm fat mass (left)
	Arm fat mass (right)
	Arm fat percentage (left)
	Arm fat percentage (right)
	Arm tissue fat percentage (left)
	Arm tissue fat percentage (right)
	Arms fat mass
	Arms tissue fat percentage
	Body fat percentage
	Gynoid fat mass
	Gynoid tissue fat percentage
	Leg fat mass (left)
	Leg fat mass (right)
	Leg fat percentage (left)
	Leg fat percentage (right)
	Leg tissue fat percentage (left)
	Leg tissue fat percentage (right)
	Legs fat mass
	Legs tissue fat percentage
	Total fat mass
	Total tissue fat percentage
	Trunk fat mass
	Trunk fat percentage
	Trunk tissue fat percentage
	Whole body fat mass
Impedance	Impedance of arm (left)
	Impedance of arm (right)
	Impedance of leg (left)
	Impedance of leg (right)
	Impedance of whole body
Reticulocyte	High light scatter reticulocyte count
	High light scatter reticulocyte percentage
	Immature reticulocyte fraction
	Mean reticulocyte volume
	Reticulocyte count
	Reticulocyte percentage
Meridian	3mm strong meridian (left)
	3mm strong meridian (right)
	3mm weak meridian (left)
	3mm weak meridian (right)

	6mm strong meridian (left)
	6mm strong meridian (right)
	6mm weak meridian (left)
	6mm weak meridian (right)
Spirometry	Forced expiratory volume in 1-second (FEV1)
	Forced expiratory volume in 1-second (FEV1), Best measure
	Forced expiratory volume in 1-second (FEV1), predicted
	Forced expiratory volume in 1-second (FEV1), predicted percentage
	Forced vital capacity (FVC)
	Forced vital capacity (FVC), Best measure
	Peak expiratory flow (PEF)

Supplementary Table 3. Phenome-wide association (PheWAS) analysis for rs114285050. Summary statistics for a stop-gain variant in *GPR151* is shown. The phenotype code used in Global Biobank Engine (GBE_code), phenotype name, N – the number of case individuals (for binary phenotypes) or individuals with non-missing values (for quantitative traits), -log10 p-value, log odds ratio, log(OR), or BETA, and 1.96 * standard error of log(OR) or BETA (1.96 * SE) are shown.

GBE code	Phenotype name	N	$-\log_{10}$ p-value	log(OR) or BETA	1.96 * SE
BIN1960	Fed-up feelings	136434	3.041	-0.09304	0.054978
INI48	Waist circumference	336659	7.599	-0.06544	0.02301
INI23100	Whole body fat mass	330970	6.87	-0.06872	0.025539
INI23128	Trunk fat mass	331295	6.835	-0.07053	0.026284
INI23120	Arm fat mass (right)	331422	6.816	-0.06863	0.025617
INI23099	Body fat percentage	331318	6.816	-0.05306	0.019816
INI23127	Trunk fat percentage	331314	6.79	-0.06356	0.023775
INI21002	Weight	336260	6.654	-0.06087	0.02303
INI23116	Leg fat mass (left)	331470	6.649	-0.05468	0.020698
INI23112	Leg fat mass (right)	331488	6.62	-0.05517	0.020933
INI21001	Body mass index (BMI)	336144	6.498	-0.06789	0.026029
INI23111	Leg fat percentage (right)	331491	6.341	-0.04201	0.016327
INI23124	Arm fat mass (left)	331362	6.317	-0.06587	0.025656
INI23115	Leg fat percentage (left)	331473	6.17	-0.04087	0.016123
INI23119	Arm fat percentage (right)	331445	5.424	-0.04689	0.019874
INI23123	Arm fat percentage (left)	331395	5.048	-0.04485	0.019796
INI49	Hip circumference	336620	4.649	-0.05669	0.026205
INI23126	Arm predicted mass (left)	331345	4.211	-0.03373	0.016499
INI23125	Arm fat-free mass (left)	331358	3.929	-0.03257	0.01658
INI23105	Basal metabolic rate	331502	3.923	-0.03368	0.017154
INI23117	Leg fat-free mass (left)	331454	3.423	-0.03063	0.016887
INI23118	Leg predicted mass (left)	331449	3.336	-0.02998	0.016776
INI23121	Arm fat-free mass (right)	331418	3.32	-0.02894	0.016241
INI23122	Arm predicted mass (right)	331413	3.176	-0.02808	0.016174
INI23102	Whole body water mass	331510	3.044	-0.02784	0.01644
INI23114	Leg predicted mass (right)	331480	3.019	-0.02812	0.016689

Supplementary Table 4. Phenome-wide association (PheWAS) analysis for rs150090666. Summary statistics for a stop-gain variant in *PDE3B* is shown. The same columns are used as in Supplementary Table 3.

GBE code	Phenotype name	N	$-\log_{10}$ p-value	log(OR) or BETA	1.96 * SE
HC269	high cholesterol	43054	4.457	-0.5904	0.279692
BIN4728	Leg pain on walking	28151	3.154	0.4366	0.252448
BIN2020	Loneliness, isolation	60153	3.098	0.2983	0.174322
INI49	Hip circumference	336620	10.75	0.2476	0.072167
INI23113	Leg fat-free mass (right)	331480	7.381	0.1293	0.046197
INI21002	Weight	336260	7.333	0.1769	0.063445
INI23114	Leg predicted mass (right)	331480	7.3	0.1276	0.045884
INI23128	Trunk fat mass	331295	7.079	0.1977	0.072304
INI23117	Leg fat-free mass (left)	331454	6.965	0.1259	0.046432
INI23118	Leg predicted mass (left)	331449	6.958	0.1249	0.046119
INI20015	Sitting height	336513	6.783	0.1454	0.054449
INI23105	Basal metabolic rate	331502	6.141	0.1193	0.047177
INI23127	Trunk fat percentage	331314	6.059	0.1641	0.065405
INI50	Standing height	336500	6	0.1266	0.050725
INI23100	Whole body fat mass	330970	5.895	0.1736	0.070227
INI23120	Arm fat mass (right)	331422	5.601	0.1692	0.070462
INI23124	Arm fat mass (left)	331362	5.255	0.1635	0.070521
INI23102	Whole body water mass	331510	5.107	0.1031	0.045198
INI23101	Whole body fat-free mass	331486	5.039	0.1021	0.045119
INI23099	Body fat percentage	331318	4.919	0.1217	0.054508
INI23123	Arm fat percentage (left)	331395	4.516	0.1158	0.054429
INI23119	Arm fat percentage (right)	331445	4.401	0.1146	0.054645
INI23116	Leg fat mass (left)	331470	4.208	0.1163	0.056918
INI23126	Arm predicted mass (left)	331345	4.189	0.09246	0.045374
INI23112	Leg fat mass (right)	331488	4.119	0.1162	0.057565
INI23125	Arm fat-free mass (left)	331358	4.061	0.09128	0.04559
INI23122	Arm predicted mass (right)	331413	3.746	0.085	0.044472
INI3062	Forced vital capacity (FVC)	309028	3.572	0.1001	0.053841
INI23130	Trunk predicted mass	331203	3.565	0.08357	0.045002
INI23129	Trunk fat-free mass	331234	3.508	0.08307	0.045158
INI23121	Arm fat-free mass (right)	331418	3.326	0.07965	0.044649
INI20151	Forced vital capacity (FVC), Best measure	255494	3.243	0.102	0.058016

Supplementary Table 5. Genetic correlation of summary statistics with different covariates. For five binary traits and five quantitative traits (type), genetic correlation is computed for two GWAS summary statistics computed with four and ten genotype principal components in the covariates. The phenotype name and its corresponding global biobank engine code (GBE code) are shown.

Type	GBE code	Phenotype name	Genetic correlation
Binary	HC382	Asthma	1.0003
Binary	HC259	High cholesterol	0.9973
Binary	cancer1003	Skin cancer	0.9992
Binary	FH1065	High blood pressure	1.0
Binary	MED1140884600	Metformin	0.9994
Continuous	INI21001	BMI	1.0
Continuous	INI50	Standing height	1.0
Continuous	INI3062	Forced vital capacity (FVC)	1.0
Continuous	INI30120	Lymphocyte count	1.0
Continuous	INI3786	Age asthma diagnosed	1.0

Supplementary References

1. DeBoever, C. *et al.* Medical relevance of protein-truncating variants across 337,205 individuals in the UK Biobank study. *Nat. Commun.* **9**, 1612 (2018).
