# Supplementary material for: Components of genetic associations across 2,138 phenotypes in the UK Biobank highlight adipocyte biology
Source: Nat Commun. 2019 Sep 6;10:4064. doi: 10.1038/s41467-019-11953-9 (PMC6731283; doi:10.1038/s41467-019-11953-9)
Supplement: Supplementary file 3 — Reporting Summary [file 41467_2019_11953_MOESM3_ESM.pdf]

nature research

Corresponding author(s): Dr. Manuel A. Rivas & Dr. Erik Ingelsson

Last updated by author(s): Aug 7, 2019

Reporting Summary

Nature Research wishes to improve the reproducibility of the work that we publish. This form provides structure for consistency and transparency in reporting. For further information on Nature Research policies, see Authors & Referees and the Editorial Policy Checklist.

Statistics

For all statistical analyses, confirm that the following items are present in the figure legend, table legend, main text, or Methods section.

n/a	Confirmed	
☐	☒	The exact sample size (*n*) for each experimental group/condition, given as a discrete number and unit of measurement
☐	☒	A statement on whether measurements were taken from distinct samples or whether the same sample was measured repeatedly
☐	☒	The statistical test(s) used AND whether they are one- or two-sided<br>*Only common tests should be described solely by name; describe more complex techniques in the Methods section.*
☐	☒	A description of all covariates tested
☐	☒	A description of any assumptions or corrections, such as tests of normality and adjustment for multiple comparisons
☐	☒	A full description of the statistical parameters including central tendency (e.g. means) or other basic estimates (e.g. regression coefficient) AND variation (e.g. standard deviation) or associated estimates of uncertainty (e.g. confidence intervals)
☐	☒	For null hypothesis testing, the test statistic (e.g. *F*, *t*, *r*) with confidence intervals, effect sizes, degrees of freedom and *P* value noted<br>*Give P values as exact values whenever suitable.*
☒	☐	For Bayesian analysis, information on the choice of priors and Markov chain Monte Carlo settings
☒	☐	For hierarchical and complex designs, identification of the appropriate level for tests and full reporting of outcomes
☐	☒	Estimates of effect sizes (e.g. Cohen's *d*, Pearson's *r*), indicating how they were calculated

Our web collection on statistics for biologists contains articles on many of the points above.

Software and code

Policy information about availability of computer code

Data collection

We used array-genotype data and phenotype data from the UK Biobank [Ref:14]. The primary data used to generate the analyses presented here are available in the UK Biobank access management system (https://amsportal.ukbiobank.ac.uk/) for application 24983, “Generating effective therapeutic hypotheses from genomic and hospital linkage data” (http://www.ukbiobank.ac.uk/wp-content/uploads/2017/06/24983-Dr-Manuel-Rivas.pdf). The list of phenotype fields used in the study is reported in Supplementary Tables 1-2.

Data analysis

See Method section for the full details of the data analysis. We used the following software for the data analysis.
- VEP LOFTEE plugin (https://github.com/konradjk/loftee)
- PLINK v2.00a (17 July 2017) and v2.00a (20 Sep. 2017) (https://www.cog-genomics.org/plink/2.0/)
- The genomic region enrichment analysis tool (GREAT) v4.0.3 (http://great.stanford.edu)
- SciDB (https://www.paradigm4.com/)
- Jupyter notebook with Python and R kernels (http://jupyter.org/)
- GraphPad Prism version 7 (https://www.graphpad.com/scientific-software/prism/)

For manuscripts utilizing custom algorithms or software that are central to the research but not yet described in published literature, software must be made available to editors/reviewers. We strongly encourage code deposition in a community repository (e.g. GitHub). See the Nature Research guidelines for submitting code & software for further information.

Data

Policy information about availability of data

All manuscripts must include a data availability statement. This statement should provide the following information, where applicable:
- Accession codes, unique identifiers, or web links for publicly available datasets
- A list of figures that have associated raw data
- A description of any restrictions on data availability

The association analysis data, the interactive DeGAs App, and its video tutorial are available as a part of Global Biobank Engine (https://biobankengine.stanford.edu/

degas). The decomposed matrices are available on figshare (https://doi.org/10.35092/yhjc.9202247). The source data underlying Figs. 2a-b, 4c, and 5 and Supplementary Figs. 27-28 are provided as a Source Data file.

Field-specific reporting

Please select the one below that is the best fit for your research. If you are not sure, read the appropriate sections before making your selection.

☒ Life sciences ☐ Behavioural & social sciences ☐ Ecological, evolutionary & environmental sciences

For a reference copy of the document with all sections, see nature.com/documents/nr-reporting-summary-flat.pdf

Life sciences study design

All studies must disclose on these points even when the disclosure is negative.

Sample size

Computational and statistical analyses:
We used the UK Biobank population cohort and focused on 337,199 White British individuals (see below).

Functional experiments:
All data were based on at least three independent experiments.

Data exclusions

Computational and statistical analyses:
To minimize the variabilities due to population structure in our dataset, we restricted our analyses to include 337,199 White British individuals based on the following five criteria reported by the UK Biobank in the file “ukb_sqc_v2.txt”:
1. self- reported white British ancestry (“in_white_British_ancestry_subset” column)
2. used to compute principal components (“used_in_pca_calculation” column)
3. not marked as outliers for heterozygosity and missing rates (“het_missing_outliers” column)
4. do not show putative sex chromosome aneuploidy (“putative_sex_chromo- some_aneuploidy” column)
5. have at most 10 putative third-degree relatives (“excess_relatives” column).
Please refer to "Genotype data preparation" subsection in Method section for more details.

Functional experiments:
No data were excluded.

Replication

All experimental findings were successfully replicated, and results reliably reproduced.

Randomization

Cells were randomly assigned to each experimental group.

Blinding

Blinding was not applicable to functional cellular experiments, as the operator needed to perform various treatments to the cells and then collect the relevant data from the cells.

Reporting for specific materials, systems and methods

We require information from authors about some types of materials, experimental systems and methods used in many studies. Here, indicate whether each material, system or method listed is relevant to your study. If you are not sure if a list item applies to your research, read the appropriate section before selecting a response.

Materials & experimental systems

n/a	Involved in the study	
☐	☒	Antibodies
☐	☒	Eukaryotic cell lines
☒	☐	Palaeontology
☒	☐	Animals and other organisms
☐	☒	Human research participants
☒	☐	Clinical data

Methods

n/a	Involved in the study	
☒	☐	ChIP-seq
☐	☒	Flow cytometry
☒	☐	MRI-based neuroimaging

Antibodies

Antibodies used

All antibody information including catalog numbers and company source are provided in the Methods section under “Western Blot Analysis”.

Validation

All antibodies used in this study were validated by the manufacturers.

Eukaryotic cell lines

Policy information about cell lines

Cell line source(s)	Mouse 3T3-L1 cells were commercially available from ATCC. Human SGBS cells were a gift from Dr. Martin Wabitsch, University of Ulm, Germany.
Authentication	3T3-L1 was authenticated by ATCC, and SGBS was authenticated by Dr. Martin Wabitsch, University of Ulm, Germany.
Mycoplasma contamination	All cells were tested negative for mycoplasma contamination.
Commonly misidentified lines (See ICLAC register)	3T3-L1 and SGBS are the most commonly used preadipocyte cell lines of rodent and human origin, respectively.

Human research participants

Policy information about studies involving human research participants

Population characteristics	We used the UK Biobank population cohort and analyzed White British cohort (n = 337,199).
Recruitment	This research has been conducted using the UK Biobank Resource under Application Number 24983, “Generating effective therapeutic hypotheses from genomic and hospital linkage data” (http://www.ukbiobank.ac.uk/wp-content/uploads/2017/06/24983-Dr-Manuel-Rivas.pdf).
Ethics oversight	Based on the information provided in Protocol 44532 the Stanford IRB has determined that the research does not involve human subjects as defined in 45 CFR 46.102(f) or 21 CFR 50.3(g).

Note that full information on the approval of the study protocol must also be provided in the manuscript.

Flow Cytometry

Plots

Confirm that:

☒ The axis labels state the marker and fluorochrome used (e.g. CD4-FITC).

☒ The axis scales are clearly visible. Include numbers along axes only for bottom left plot of group (a 'group' is an analysis of identical markers).

☒ All plots are contour plots with outliers or pseudocolor plots.

☒ A numerical value for number of cells or percentage (with statistics) is provided.

Methodology

Sample preparation	Sample preparation was described in Methods section under “Flow Cytometry Analysis”.
Instrument	BD InfluxTM Cell Sorter was used for cell sorting.
Software	FlowJo V10 software was used to analyze flow cytometry data.
Cell population abundance	Cell population abundance was listed in Supplementary Figure 27 l.
Gating strategy	Gating strategy was shown in Supplementary Figure 27 g-l.

☒ Tick this box to confirm that a figure exemplifying the gating strategy is provided in the Supplementary Information.
