# Supplementary material for: Components of genetic associations across 2,138 phenotypes in the UK Biobank highlight adipocyte biology
Source: Nat Commun. 2019 Sep 6;10:4064. doi: 10.1038/s41467-019-11953-9 (PMC6731283; doi:10.1038/s41467-019-11953-9)
Supplement: Supplementary file 4 — Description of Additional Supplementary Files [file 41467_2019_11953_MOESM4_ESM.pdf]

Description of Additional Supplementary Files

Supplementary Data 1. List of phenotypes.
The list of phenotypes considered in the study. The table is sorted by category, number cases (for binary phenotypes), and the number of non-missing values (for quantitative phenotypes). The two columns, "All", “Coding”, and "PTVs" indicates whether the phenotype is used in each of the datasets after imposing the filters on the genome-and phenome-wide summary statistics matrix. One can browse the summary statistics from genome-wide association studies on the Global Biobank Engine with the URL in the table.

Supplementary Data 2. Summary of contribution scores for the key components.
The list of top 20 driving phenotypes, genes, and variants for the first five principal components and the top three key components for the phenotypes highlighted in the study are summarized in the table.

Supplementary Data 3. GREAT enrichment analysis for BMI.
Biological characterization of driving non-coding and coding variants of the key components for BMI with the genomic region enrichment analysis tool (GREAT) using the all variants dataset. The results of the enrichment analysis for MGI phenotype ontology, a manually curated genotype-phenotype relationship knowledgebase for mouse, is summarized by the key components. The two major summary statistics from GREAT, binomial fold and binomial p-value, are shown. Abbreviation. BFold: binomial fold, BPval: binomial p-value.

Supplementary Data 4. GREAT enrichment analysis for MI.
Biological characterization of driving non-coding and coding variants of the key components for MI with the genomic region enrichment analysis tool (GREAT) using the all variants dataset. The results are shown in the same format as in Supplementary Data 3.

Supplementary Data 5. GREAT enrichment analysis for gallstones.
Biological characterization of driving non-coding and coding variants of the key components for gallstones with the genomic region enrichment analysis tool (GREAT) using the all variants dataset. The results are shown in the same format as in Supplementary Data 3.
